# Widespread occurrence of asexual reproduction in higher termites of the *Termes* group (Termitidae: Termitinae)

**DOI:** 10.1186/s12862-019-1459-3

**Published:** 2019-06-21

**Authors:** Simon Hellemans, Klára Dolejšová, Jan Křivánek, Denis Fournier, Robert Hanus, Yves Roisin

**Affiliations:** 1 Evolutionary Biology & Ecology, Université Libre de Bruxelles, Avenue F.D. Roosevelt 50, CP 160/12, B-1050 Brussels, Belgium; 2 Chemistry of Social Insects, Institute of Organic Chemistry and Biochemistry of the Czech Academy of Sciences, Flemingovo n. 2, CZ-166 10 Prague 6, Czech Republic

**Keywords:** Asexual queen succession, Thelytokous parthenogenesis, Gamete duplication, *Termes*-group, South America, Termites

## Abstract

**Background:**

A decade ago, the mixed reproductive strategy Asexual Queen Succession (AQS) was first described in termites. In AQS species, the workers, soldiers and dispersing reproductives are produced through sexual reproduction, while non-dispersing (neotenic) queens arise through automictic thelytokous parthenogenesis, replace the founding queen and mate with the founding king. As yet, AQS has been documented in six species from three lineages of lower (Rhinotermitidae) and higher (Termitinae: *Termes* group and Syntermitinae) termites. Independent evolution of the capacity of thelytoky as a preadaptation to AQS is supported by different mechanisms of automixis in each of the three clades. These pioneering discoveries prompt the question on the extent of thelytoky and AQS in the diversified family of higher termites.

**Results:**

Here, we investigated the capacity of thelytoky and occurrence of AQS in three species from the phylogenetic proximity of the neotropical AQS species *Cavitermes tuberosus* (Termitinae: *Termes* group): *Palmitermes impostor*, *Spinitermes trispinosus*, and *Inquilinitermes inquilinus*. We show that queens of all three species are able to lay unfertilized eggs, which undergo thelytokous parthenogenesis (via gamete duplication as in *C. tuberosus*) and develop through the transitional stage of aspirants into replacement neotenic queens.

**Conclusions:**

The breeding system in *P. impostor* is very reminiscent of that described in *C. tuberosus* and can be characterized as AQS. In the remaining two species, our limited data do not allow classifying the breeding system as AQS; yet, also in these species the thelytokous production of neotenic females appears to be a systematic element of reproductive strategies. It appears likely that the capacity of thelytokous parthenogenesis evolved once in the *Termes* group, and may ultimately be found more widely, well beyond these Neotropical species.

**Electronic supplementary material:**

The online version of this article (10.1186/s12862-019-1459-3) contains supplementary material, which is available to authorized users.

## Background

Nearly a decade ago, a unique reproductive strategy, dubbed Asexual Queen Succession (AQS), was described for the first time in termites [1]. The founding queens of AQS species produce the sterile colony members as well as (most) dispersing reproductives through conventional sexual process from eggs fertilized by the founding king. In addition, the queens also lay unfertilized eggs that undergo automictic thelytokous parthenogenesis and are destined to develop through a series of nymphal stages into replacement neotenic queens. These non-dispersing queens, present sometimes in large numbers reaching up to several hundreds, replace their mother at some moment of the colony development and mate with the founding king, making up a “harem” breeding structure typical for AQS. The combination of sexual and asexual processes maximizes the genetic input of the queen(s) into the next generation of non-dispersing queens while conserving a high genetic diversity in sterile helpers (workers and soldiers) and dispersers (future kings and queens). Ultimately, AQS brings two main advantages at the colony level: (*i*) it increases the reproductive potential of the colony through the replacement of one founding female by multiple queens bearing an undiluted gene pool of the founding queen, and (*ii*) it extends the colony lifespan due to the ability of the replacement queens to produce subsequent generations of parthenogens developing into neotenic queens, leading to a virtual “genetic immortality” of the foundress (reviewed in [2]).

The discovery of AQS in termites represented a fascinating example of convergent evolutionary processes leading to mixed modes of reproduction, combining the benefits of asexual and sexual reproduction, in both major groups of social insects, the ants and the termites. In ants, an analogous strategy alternating thelytoky for the production of new generations of queens with the sexual production of workers is known in several species (reviewed in [3]). The capacity for thelytokous parthenogenesis was recorded in the past in several termite species from different families, but it has long been disregarded as an accidental event or emergency strategy used by foundresses that fail in encountering a partner (reviewed in [2, 4]) and the parthenogenetic offspring often has low survival rates [5, 6]. Only in a handful of species, the preadaptation to thelytoky is now known to have evolved into a crucial and stable element of the colony lifecycle, giving rise to AQS breeding system. AQS was first described in three species of the temperate wood-feeding subterranean lower termites from the genus *Reticulitermes* (Rhinotermitidae) [1, 7, 8] and was thus for some time considered as a singularity restricted to this genus, even though the three AQS species are not close relatives within the genus *Reticulitermes* [9]. More recently, we uncovered AQS in three neotropical species of soil-feeding higher termites (Termitidae), remote from *Reticulitermes*. Two cases were identified in the subfamily Syntermitinae, i.e. *Embiratermes neotenicus* [10] and *Silvestritermes minutus* [11]. The two species are rather distant within Syntermitinae and separated by a number of species with no record of a harem breeding structure [12]. One additional occurrence of AQS has been documented in *Cavitermes tuberosus* from the subfamily Termitinae [13]. Thus, the current knowledge suggests at least six independent origins of the AQS breeding system in termites.

The independent evolution of AQS in the three lineages (Rhinotermitidae, Termitinae, Syntermitinae) is evident also from fundamental differences in the mode of ploidy restoration following the meiotic process during the development of thelytokous eggs, i.e. terminal fusion in *Reticulitermes*, central fusion in Syntermitinae, and gamete duplication in *C. tuberosus*. While having an impact on the frequency of homozygous genotypes in the parthenogens (almost complete or full loss of heterozygosity under terminal fusion and gamete duplication, respectively, vs. high conservation rate of maternal heterozygosity under central fusion), the different cytogenetic mechanisms do not change the global pattern of gene flow proper to AQS at the colony level. Therefore, the main genetic advantages of the independently evolved AQS cases remain qualitatively comparable in all lineages. At the same time, individual AQS species differ in quantitative modalities of their life cycles and breeding systems, such as the timing of primary queen replacement or the average numbers of parthenogens to replace the founding queen, suggesting that different species may use differently the benefits offered by AQS [2, 11].

The recent descriptions of three AQS species from two subfamilies of Termitidae in the Neotropics prompt the question on the extent of this outstanding strategy across the phylogenetic diversity of higher termites. The three species represent soil-feeding termites inhabiting rather conspicuous epigeous nests, whose breeding structure can be, upon careful inspection, thoroughly studied. In fact, the harem structure was previously noted in descriptive reports on the two known AQS cases in Syntermitinae [14–16]. In many other tropical soil feeders, the nesting habits are more cryptic, their nests often subterranean and/or diffuse, and the royal chambers difficult to access. Because the presence of AQS cannot be identified based on the genotypes of sterile castes only, the breeding system in many tropical species remains largely unknown.

Therefore, we investigated the capacity for thelytoky and the possible occurrence of AQS in the phylogenetic proximity of the genus *Cavitermes*. In mature colonies of *Cavitermes tuberosus*, the founding primary queen gets replaced by large harems of non-physogastric neotenic queens (up to 667). The majority of these neotenic queens (82%) bear only maternal alleles and are homozygous at all loci, which suggests their origin through thelytokous parthenogenesis via automixis with gamete duplication [13]. They develop from nymphs of the third and fourth stage through a transitional subfertile stage of “aspirants” [17] and reproduce with the founding primary king. In comparison with AQS cases in Syntermitinae, the replacement of the foundress by parthenogens in *C. tuberosus* takes place rather late in the colony life cycle. While in the syntermitines *E. neotenicus* and *S. minutus*, the queen replacement is an obligatory event preceding the colony maturation and production of dispersers [10, 11], the primary queens of *C. tuberosus* are often replaced only after the colony reaches maturity and already produces dispersing reproductives [13, 18]. Therefore, the total incidence of the harem breeding structure is lower in *C. tuberosus* (44% of mature colonies) and the presence of AQS can be viewed as a tool to extend the colony’s reproductive potential and lifespan beyond those of the foundress in late stages of colony existence [13].

While the general relationships inside the paraphyletic subfamily Termitinae are rather poorly resolved, *Cavitermes* belongs to a well-defined branch of Neotropical genera, supported by several molecular studies and referred to as the *Cavitermes* lineage [19–21]. It contains the genera *Spinitermes* Wasmann, 1897 (5 spp.), *Dihoplotermes* Araujo, 1961 (2 spp.), *Cavitermes* Emerson, 1925 (5 spp.), its newly described sister genus *Palmitermes* Hellemans & Roisin, 2017 (1 sp.), and probably also the genus *Divinotermes* Carrijo & Cancello, 2011 (3 spp.) [21–24]. Beside the detailed study on *C. tuberosus*, the data on the breeding systems in the remaining genera are scarce. However, in contrast to the general rare incidence of neotenic reproductives in higher termites [25], there are several reports on the presence of neotenics of nymphal origin in the genera from the *Cavitermes* lineage, namely in *Palmitermes impostor* [21], *Spinitermes brevicornutus*, *S. nigrostomus* [26], *S. robustus* and *S. trispinosus* [27]. Here, we report on the breeding structures, occurrence of neotenic reproductives and genetic structure of colonies with special emphasis on the eventual presence of thelytokous parthenogenesis in reproductive strategies of three Neotropical species from the *Termes*-group (sensu [28]) of the Termitinae subfamily, i.e. *Palmitermes impostor* and *Spinitermes trispinosus* from the *Cavitermes* lineage, and *Inquilinitermes inquilinus* from the sister *Termes* lineage.

## Results

### Microsatellite polymorphisms

Global tests of genotypic linkage disequilibrium (LD) revealed no departure for any combination of loci in *I. inquilinus* and *P. impostor*. For *S. trispinosus*, due to limited available data and genetic diversity of markers, LD tests performed on datasets consisting of one worker per nest were not possible. When pooling all workers (*n* = 31) to one population, LD was significant only for the pair *Ctub*-72/*Ctub*-95 (*p* = 0). Hardy-Weinberg exact tests revealed that all loci, for all species, were at HWE. No sign of null alleles was detected at the population level for none of the species, except at loci *Ctub*-21 and *Ctub*-72 in *P. impostor* (frequency of 0.07 and 0.08 according to the Brookfield 1 estimator, respectively) as suggested by the general excess of homozygotes. There was no evidence of scoring error due to large allelic dropout.

The observed and effective number of alleles ranged from 2 to 7 and 1.61 to 3.12 in *P. impostor* (*n* = 92 workers), 3 to 5 and 1.58 to 4.31 in *S. trispinosus* (*n* = 31), and 2 to 6 and 1.36 to 3.56 in *I. inquilinus* (*n* = 36; Additional file 1: Table S2). Observed heterozygosities ranged from 0.078 to 0.730 and expected heterozygosities from 0.075 to 0.680 in *P. impostor*, 0.452 to 0.793 and 0.367 to 0.768 in *S. trispinosus*, 0.294 to 0.943 and 0.251 to 0.719 in *I. inquilinus* (Additional file 1: Table S2).

### *Parthenogenesis and asexual queen succession in *P. impostor

**Fig. 1:**
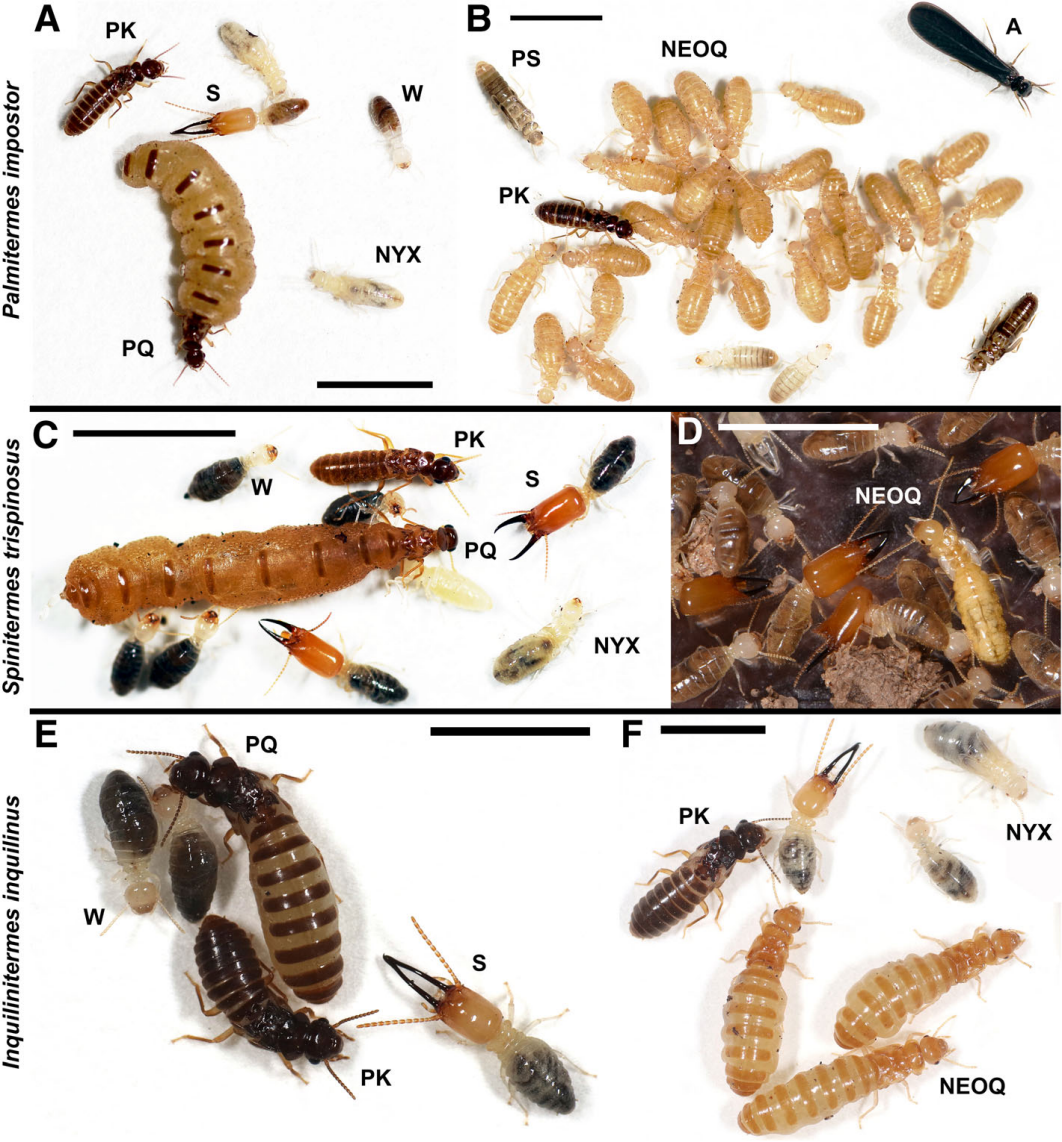
Photographs of reproductives of *Palmitermes impostor* (**a**, **b**), *Spinitermes trispinosus* (**c**, **d**) and *Inquilinitermes inquilinus* (**e**, **f**). Primary colonies (**a**, **c**, **e**) are headed by a primary king (PK) and a primary queen (PQ), and secondary colonies (**b**, **d**, **f**) are headed by numerous nymphoid neotenic queens (NEOQ). Neotenics develop from a nymphal stage called aspirant (NYX) displaying a soil-filled digestive tube and translucent wing buds. Female aspirants and neotenics are mainly produced through thelytoky while the dispersing reproductives (alates, A) and sterile castes, workers (W) and soldiers (S), are produced through sexual reproduction. Additional female reproductives, two pseudoimagos (PS) with incomplete imaginal pigmentation and only partially developed wings, were found in *P. impostor* (**b**). Scale bars, 5 mm

Social composition of all sampled colonies is summarized in the (see Additional file 1: Table S1). Reproductives of at least one sex were found in 19 nests. Thirteen colonies (68%) were classified as primary colonies headed by primary reproductives, with no indication of queen or king replacement. In 11 of them, we succeeded in finding the complete primary pair; in the remaining two only one of the primaries was found. By contrast, six other nests (32%) were categorized as secondary colonies after primary queen replacement, because they contained one to 129 nymphoid neotenic queens and no primary queen. In three of these colonies, we also succeeded in finding one primary king (Fig. 1a,b; see also Additional file 1: Table S1). No male reproductives other than a single primary king were recorded in any of the 19 nests. The majority of the neotenic queens were physogastric and displayed a deep ochre tinge, while a few of them were greyish and non-physogastric, seemingly before full maturation (Fig. 1b). Additional female reproductives of two types were found in three nests: (*i*) two pseudoimagos with incomplete imaginal pigmentation and only partially developed wings found in one nest (Fig. 1b), and (*ii*) one ergatoid (worker-derived) neotenic showing fully developed compound eyes and no traces of wing buds, found in two nests (Additional file 1: Figure S1). From 26 nests, we collected nymphs of stages 1 to 4. Among them, we distinguished in 23 nests peculiar female nymphs of the fourth stage, corresponding by their phenotype to the aspirants (or preneotenics, prior to the development into fertile neotenic queens) previously described in *C. tuberosus* [17]. Female aspirants were found in numbers of one to 232, and as in *C. tuberosus*, they displayed soil-filled digestive tube and translucent wing buds (Fig. 1a). Thousands of future dispersers (nymphs of the fifth stage and/or alates) were collected from five nests in June 2016 (three were headed by primary reproductives, two by a primary king and a harem of neotenic queens), and a few were found in one nest collected in January 2017, indicating that dispersal flights mainly occur at the end of the principal wet season.

**Fig. 2:**
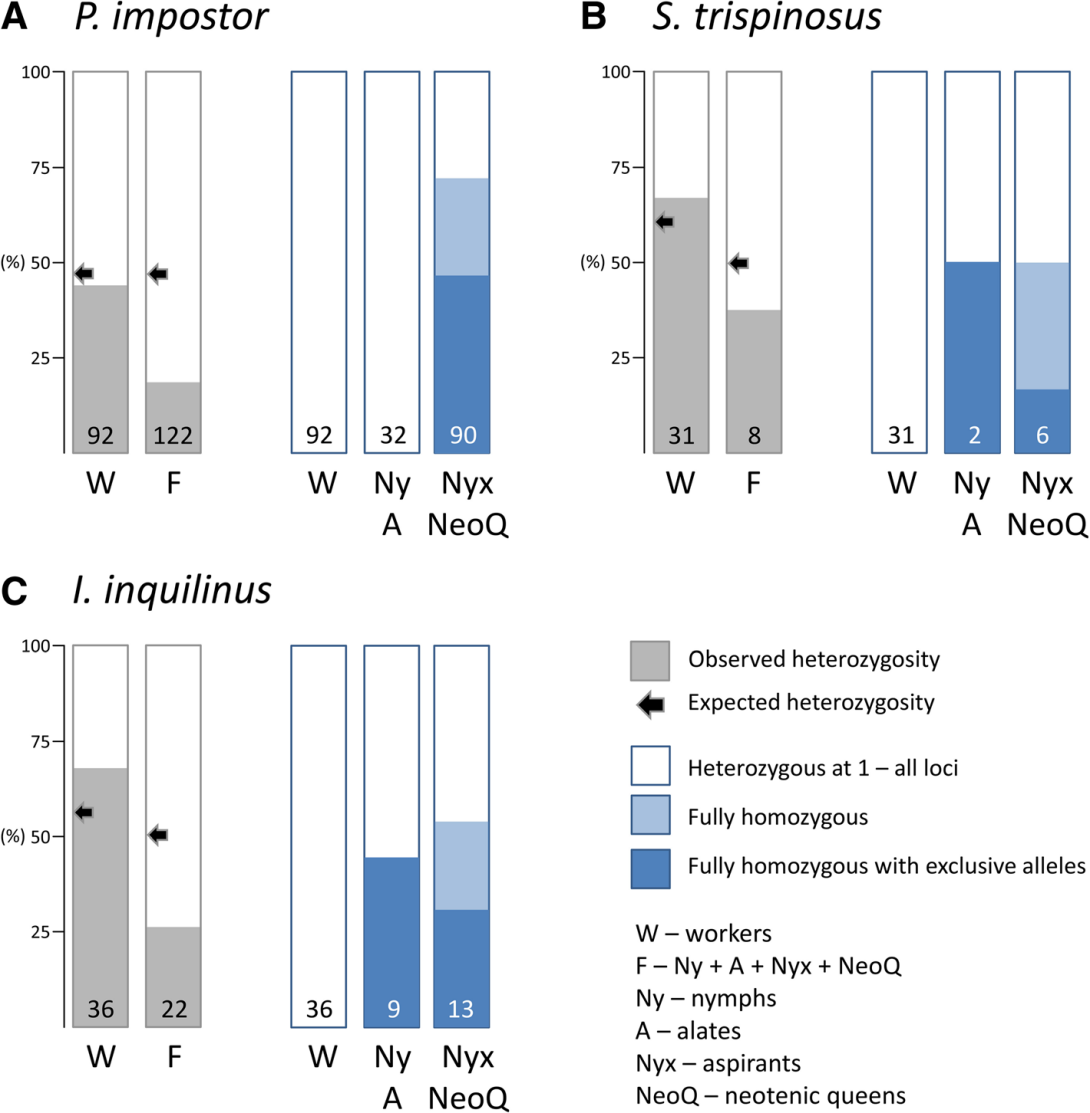
Observed and expected heterozygosities (left panel) and proportions of heterozygous and fully homozygous (with or without exclusive alleles of one founding reproductive) individuals (right panel) in workers and females of the nymphal line in *P. impostor* (**a**), *S. trispinosus* (**b**) and *I. inquilinus* (**c**). Numbers represent the number of genotyped individuals

When taking into account all individuals, the nine analysed microsatellite loci in the 11 genotyped colonies displayed a number of alleles ranging from 2 to 7 (mean ± SD = 3.11 ± 1.76). In all colonies and at all loci, we detected a maximum of four alleles, suggesting that they were initiated by a single pair of reproductives (Additional file 1: Table S3). Overall, the mean number of effective alleles over nest and loci (1.66 ± 0.05) was lower than the mean number of observed alleles (1.93 ± 0.06; paired *t*-test, *t* = 6.006, *df* = 8, *p* < 0.001), indicating uneven allelic segregation. The worker caste displayed a higher observed heterozygosity than females from the nymphal line (nymphs, aspirants and neotenic queens) (0.44 ± 0.06 vs 0.18 ± 0.03; paired *t*-test, *t* = 7.617, *df* = 8, *p* < 0.001; Fig. 2a).

Eight of the genotyped colonies (PA–PH) were classified as “primary colonies” based on the social composition, because we did not find any mature reproductives additional to the observed or presumed primaries. Each of them only contained groups of subfertile female aspirants (i.e., future neotenic queens). Sterile castes and male nymphs showed a maximum of four genotypes consistent with their origin as direct offspring of the colony founders, confirming the presumed simple family design.

The genotype patterns were more complex for the females from the nymphal line in these eight colonies. While in all females retrieved as classical nymphs, the genotypes were similar to those of the sterile castes and suggested they are offspring of colony founders, the genotypes of the 49 female aspirants split in two categories (Fig. 2a). The first one contained seven females heterozygous at two to five loci, suggesting their sexual origin. In five of them, the genotypes matched those of sterile castes and male nymphs, while the other two (from the colony PG) were homozygous at the locus *Ctub*-74 for a presumably paternal allele, which may be due to the intervention of an unsampled generation of sexually produced neotenic females. The remaining 42 female aspirants were fully homozygous, showed a maximum of two genotype classes per locus per colony, and possessed the alleles exclusive to the primary queen or to one of the two inferred primaries, at one to five loci, while lacking the exclusive alleles of the primary male (Additional file 1: Table S3). This indicates that these future neotenic queens were produced through automictic thelytokous parthenogenesis, most likely through gamete duplication, as observed previously in *C. tuberosus*.

The remaining three colonies (PI–PK) were classified as secondary colonies after primary queen replacement, because they contained groups of mature neotenic queens, either in combination with a primary king (2 cases) or no male was retrieved (1 case). In addition, one ergatoid neotenic female was observed in the colony PJ (Additional file 1: Figure S1). Beside the group of neotenic queens, two of the three colonies also contained a generation of female aspirants, presumed offspring of the present neotenic queens. The genotypes of the sterile castes showed allelic combinations and/or genotypic frequencies inconsistent with simple family design. Their uneven distribution likely resulted from inbred matings involving sexually produced neotenics. Indeed, a portion of the mature neotenic queens in all three colonies was of sexual origin (see below), confirming thus the extended family pattern due to father-daughter inbreeding between the primary king and his sexually produced neotenic daughters, giving rise to the genotyped offspring (sterile castes, alates).

The reproductive origin of females from the nymphal line in these three colonies was mixed. Out of the 24 neotenic females, 16 were apparently of sexual origin with one to six heterozygous loci in their genotypes and two to four genotypes per locus and colony. The remaining eight female neotenics were homozygous at all loci, one of them lacking paternal alleles at two loci (Fig. 2a; Additional file 1: Table S3). Even though we were not able to reconstruct completely the genotypes of both colony founders, the full homozygosity of these neotenic queens is analogous to that of the aspirant females with unambiguous parthenogenetic origin, reported above in the eight simple families. Fifteen out of the 16 female aspirants (from colonies PJ and PK) were also fully homozygous. From them, all eight aspirants in the colony PK lacked the alleles exclusive to the genotyped primary king at two to four loci per individual, which provides a direct support for their thelytokous origin. All eight genotyped alate females from the colony PK and the ergatoid neotenic female were of sexual origin.

### *Occurrence of parthenogenesis in* S. trispinosus

Primary reproductives were collected from five nests of *S. trispinosus*, one nymphoid neotenic queen from two other nests, and nymphs of stages 1 to 4 from five nests (Fig. 1c, d). Among the female nymphs, we distinguished female aspirants present in three colonies (Fig. 1c; Additional file 1: Table S1). Nymphs of the fifth stage were found in two nests collected in April 2017 and two nests from June 2016, indicating that the dispersal flights take place at the end of the wet season.

When including all individuals from the four genotyped nests, the three microsatellite loci displayed a number of alleles ranging from three to five (mean ± SE = 4.00 ± 0.58). The workers in all nests exhibited a maximum of three alleles and four genotypes per locus, and where the primaries were known, the genetic constitution of workers matched with the parental genotypes (Additional file 1: Table S4). This indicated the breeding pattern of all colonies to be the simple family.

For the total dataset from all nests, the mean number of effective alleles (1.92 ± 0.15) was not different from the mean number of observed alleles (2.33 ± 0.14; Wilcoxon signed rank test, *W* = 6, *p* = 0.25). Observed heterozygosities did not differ between workers and females (0.67 ± 0.11 vs. 0.38 ± 0.00; Wilcoxon signed rank test, *W* = 6, *p* = 0.25; Fig. 2b). From the eight genotyped females of the nymphal line, four were homozygous at all loci. For three of them found in the nest SD (two aspirants and one nymph), the probability of their sexual origin was of 0.063, based on the inferred genotypes of the founders. One fully homozygous female aspirant found in combination with the primary reproductives in the colony SA lacked the paternal alleles, which unambiguously indicated its origin via thelytokous parthenogenesis. The full homozygosity also suggests that the ploidy is likely to be restored through gamete duplication. In addition, the primary queen heading the colony SD was also fully homozygous. Based on population allelic frequencies, its probability to be sexually produced was low (*p* = 0.008).

### *Occurrence of parthenogenesis in* I. inquilinus

Reproductives of at least one sex were found in 14 nests of *I. inquilinus*. Among the nests containing neotenics, 1–10 neotenic queen(s) and 1–2 neotenic king(s) were retrieved. The breeding structure was overall quite diversified (Fig. 1e, f; Additional file 1: Table S1): four colonies were primary-headed, two harboured both the primary queen and king as well as neotenic king(s) and queens, two contained the primary king in combination with neotenic king and/or queens, two contained only female neotenics, and a single one neotenics of both sexes. Finally, two nests collected in April 2018 contained, beside a pair of mature primaries, also several young dealated primary males and females. Nymphs of the fifth stage and/or alates were found in nests collected in October 2014, April 2017, and April–May 2018.

When taking into account all individuals from the five genotyped nests, the eight microsatellite loci displayed two to seven alleles (mean ± SE = 2.43 ± 0.13), with a maximum of four alleles per locus and colony, suggesting biparental origin of the colonies (Additional file 1: Table S5). The mean number of effective alleles (1.97 ± 0.09) was lower than the mean number of observed alleles (2.38 ± 0.12; Wilcoxon signed rank test, *W* = 36, *p* = 0.008), indicating uneven allelic segregation. The workers displayed a higher observed heterozygosity than females from the nymphal line (0.68 ± 0.08 vs. 0.26 ± 0.04; Wilcoxon signed rank test, *W* = 36, *p* = 0.008; Fig. 2c).

Four colonies (IA–ID) exhibited a maximum of four genotypes per locus in workers, male nymphs and/or male aspirants. These genotypes were consistent with the parental ones or allowed a reconstruction of a parental pair of genotypes, giving support to the simple family design. One female nymph out of four genotyped, all three aspirants and one out of two neotenic queens were fully homozygous and displayed alleles present in only one of the parental genotypes at two to four loci (maternal genotype when the primary queen was genotyped; Fig. 2c). This indicates that these females were produced through thelytokous parthenogenesis, most likely via gamete duplication. In addition, the primary queen heading the colony ID was fully homozygous. Based on population allelic frequencies, its probability to be sexually produced was very low (*p* < 0.001).

In the remaining colony (IE), the workers showed five genotypes at locus *Iinq*-15 and all markers showed significant inbreeding (*F*_*IC*_ = 0.226), which is compatible with the occurrence of multiple sexually produced neotenic queens in the nest. The genotypes of male nymphs contained additional alleles to those found in workers (loci *Iinq*-12, *Iinq*-14 and *Iinq*-16), but not increasing the total number per locus over four. When combined with the genotypes of workers, these allelic combinations pointed once again towards the extended family design. Three out of five female nymphs and three out of eight neotenic queens from this colony were fully homozygous (Fig. 2c). Thus, even though we could not fully reconstruct the genotypes of their parents due to the extended family structure, these females were very likely of thelytokous origin, as shown above for the nymphs, aspirants and neotenic queens from the colonies IA–ID under the simple family design.

In summary, 11 out of 22 (50%) genotyped females from the nymphal line (nymphs, aspirants, neotenic queen; Fig. 2c) together with one founding primary queen appear to be produced through thelytokous parthenogenesis, probably via automixis with gamete duplication.

## Discussion

Our results demonstrate that queens of three species of South American Termitinae from the *Termes* group (*Palmitermes impostor*, *Spinitermes trispinosus* and *Inquilinitermes inquilinus*) are able to lay unfertilized eggs, which undergo thelytokous parthenogenesis and are destined to develop into replacement neotenic queens through a series of nymphal stages and a special transitional stage of aspirants [17]. Because they are completely homozygous, the ploidy restoration during the parthenogenetic process most likely proceeds through automixis with gamete duplication, as we reported in *C. tuberosus* [13]. However, it is to be noted that rare events of crossing over may occur under terminal fusion [1, 7]. Therefore, the formal determination of the mode of ploidy restoration would either require more samples and microsatellite markers, in the case of *S. trispinosus* and *I. inquilinus*, or be confirmed by cytological approaches. The significance of the asexual production of neotenic females in the three species is discussed below.

### *AQS in* Palmitermes impostor

The conditional parthenogenesis observed in *P. impostor* appears to be a stable element of the breeding system of this species, which complies with the definition of the mixed reproductive strategy known as asexual queen succession (AQS). Under this strategy, the founding primary queen gets replaced by multiple neotenic queens, which are mainly produced parthenogenetically, while members of the sterile castes and alate dispersers are produced through sexual reproduction [2]. The colony genetic structure remains unaltered after the primary queen replacement as long as the offspring are produced from the mating of the primary king with primary queen parthenogens. However, when sexually produced neotenic queens occur in the harem of replacement females and mate with the primary king or when the king itself is replaced by sexually produced neotenic son(s), the genetic contribution of both initial founders in the offspring becomes unequal, which leads to partially inbred extended family design [18, 29].

The breeding system of *Palmitermes impostor* shares the basic characteristics with that recently described in *C. tuberosus* [13], a species from its sister genus within the *Cavitermes* lineage [21]. Our field and genetic data demonstrate that colonies of *P. impostor* undergo a replacement of the founding primary queen by a harem of neotenic queens (up to 129), 33% being parthenogens of the foundress. These neotenic queens develop from the subfertile stage of aspirants, characterized by translucent wing buds and soil-filled digestive tube; this latter feature points at a different feeding behaviour and indicates that they can participate in the work tasks of the colony, as in *C. tuberosus* [17]. In some colonies, two generations of replacement females were found, i.e. a group of mature neotenic queens and a group of aspirants, very likely the offspring of the present neotenics. The majority of replacement females (Fig. 2a; 73%, of which 8/24 neotenic queens and 57/65 female aspirants) were produced through thelytokous parthenogenesis via automixis with gamete duplication, similarly to what was observed in *C. tuberosus* (85%; [13, 17]). As a certain proportion of neotenic queens are sexually produced, the observed genetic structure of old colonies may appear as extended families (e.g. nests PI–PK). Our field data also show that 32% of nests are neotenic-headed (37% in *C. tuberosus*; [13]), and that both primary and secondary colonies reach maturity (i.e. produce alates) in both species [18]. This indicates that the replacement of the primary queen takes place late in the two species, and that AQS can be viewed as a tool to extend the colony’s reproductive potential and lifespan beyond that of the foundress in late stages of the colony’s existence.

In spite of the basic similarities in the breeding systems of *P. impostor* and *C. tuberosus*, the two species differ in some life history traits. Neotenic queens of *P. impostor* show a moderate to high level of physogastry (Fig. 1a, b) and can be found in multiple royal chambers of de novo built nests; in contrast, nests of *C. tuberosus* do not display tailor-made royal chambers, and queens, non-physogastric, circulate through a network of small galleries at the margin of its host’s nest [13, 17, 21]. The absence of physogastry and the high mobility of *C. tuberosus* queens may constitute key features of the inquiline nesting strategy.

### *Occurrence of parthenogenesis in* Spinitermes trispinosus

Our sampling of *S. trispinosus* is limited as this species exhibits a cryptic nesting: the colonies are found mostly in underground nests built by other species, and their distribution is diffuse throughout the host nest. This is similar to *C. tuberosus* but the colonies of the latter species are seemingly more populous, and because they inhabit arboreal nests which are often devoid of other termite species, they are easier to collect [13]. Nevertheless, our field data show that female aspirants and neotenic queens (Fig. 1c, d) occur naturally in the studied *S. trispinosus* population. Our results also show that *S. trispinosus* queens are able to lay unfertilized eggs which develop into nymphs with a developmental priority to become aspirants and neotenic queens: three female nymphs and one aspirant were homozygous at all loci and the latter (for which parental genotypes were known) was lacking the paternal alleles, which unambiguously indicates its origin via thelytokous parthenogenesis. Furthermore, our results show that one primary queen (from nest SD) was likely parthenogenetically produced. This indicate that they experience a survivorship similar to that of sexually produced ones, as previously reported for *C. tuberosus* [13, 18].

Our data do not allow us to conclude that primary queens are systematically replaced by their parthenogenetic daughters, and that the overall breeding system can be classified as AQS. However, neotenic queens were frequently observed in four out of the five species of the genus *Spinitermes*: in *S. trispinosus*, *S. robustus* [27], *S. nigrostomus* and *S. brevicornutus* [26]. Although the authors do not mention the presence of a primary king mating with these neotenic queens in a harem-like organization, the widespread occurrence of female neotenics across the genus and our finding that *S. trispinosus* is capable of thelytokous production of female aspirants and neotenics suggest that *Spinitermes* is a candidate for the evolution of AQS.

### *Parthenogenesis in* Inquilinitermes inquilinus

Species of the genus *Inquilinitermes* are well known to be obligatory inquilines. They live inside the nests built by the termites of the genus *Constrictotermes* [30] and feed on the nest material [31, 32]. Their lifestyle possibly leads to conflicts with the host. Cohabitation success is mainly achieved through the occupation of different network of galleries within the nest, diet segregation, and mutual avoidance mediated by chemical signals [30, 33, 34]. Yet, the close cohabitation with the host may be expected to create selective pressure on the breeding system so as to prevent eventual elimination of the reproductives by the host and thus mechanisms of king and queen replacement are likely to occur is these species [25, 35]. In addition, our field observations also show that the host nest itself is little resistant to abiotic factors (heavy rainfalls), which may lead to the fragmentation of the nest and its population, another factor promoting the occurrence of replacement neotenics. However, neotenics were not recorded as yet from any of the four species of the genus *Inquilinitermes* [23, 25, 36, 37]. Therefore, we report here for the first time the occurrence of neotenic queens in *I. inquilinus* (Fig. 1f).

*Inquilinitermes inquilinus* is an obligatory inquiline species strictly associated with the arboreal nests of *C. cavifrons*, and mostly found in the bottom part of the nest. Our results show that 50% of genotyped females from the nymphal line are fully homozygous, and most likely produced through parthenogenesis via gamete duplication. Furthermore, our genetic analyses suggest that one primary queen was parthenogenetically produced, which is similar to what is observed in *C. tuberosus* [13] and *S. trispinosus*.

Field observations show that the breeding system in *I. inquilinus* is quite diverse. Neotenic queens (up to 10) were found in 50% of the nests where reproductives could be collected (14 nests), and co-occurred with a primary king in two of them in a harem typical of the AQS system. In addition, four neotenic kings were retrieved from three nests: in combination with the primary reproductives and a neotenic queen, a primary king and neotenic queens, and with neotenic queens only.

Our data show that nymphs of both sexes are present throughout the year in *I. inquilinus* colonies, which is consistent with observations in *I. fur* and *I. microcerus* [32, 36]. It is hypothesized that the colonies of these inquilines are ready to disperse at any time likely because of the low stability of the inhabited nest and risky co-habitation with the host. On the contrary, most other termites generally disperse once a year through massive flights synchronized by climatic factors [11, 38, 39]. Our data also show that several pairs of dealates can colonize one host nest (Additional file 1: Table S1), as is also the case in *C. tuberosus* [13]. Both species differ in the range of hosts: *C. tuberosus* is an inquiline of a large spectrum of arboreal nesting termites while *I. inquilinus* is the obligatory inquiline of a single species, which offers fewer opportunities of colonization for the latter and the local intraspecific competition over nesting resources may be another factor promoting the evolution of replacement strategies.

More data is required to characterize the dispersal and breeding system of *I. inquilinus*. Nevertheless, it is apparent that the nesting instability linked with the life style of this obligatory inquiline is accompanied by flexible reproductive strategies, in which the replacement of colony founders is frequent and includes the parthenogenetic production of female reproductives.

### *Evolution of parthenogenesis and AQS in the* Termes *group*

**Fig. 3:**
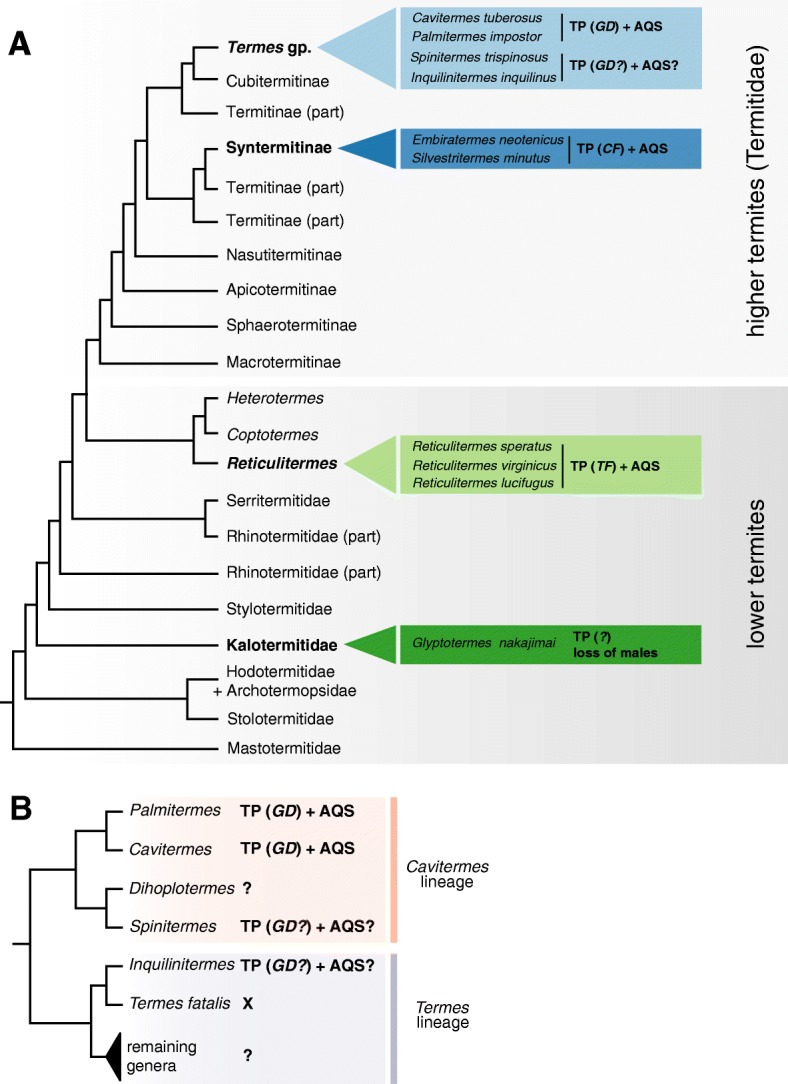
Records of frequent occurrences of thelytokous parthenogenesis in natural populations of termites, mapped on a schematic and simplified phylogenetic tree (**a**), including a detailed view on phylogenetic relationships within the *Termes* group, studied here (**b**). These trees are compiled from phylogenetic reconstructions using full mitochondrial genomes [21, 41–43]. Current knowledge suggests that AQS evolved at least six times independently —three times in the genus *Reticulitermes*, twice in the Syntermitinae and at least once in the *Termes* group. The independent evolution of thelytokous parthenogenesis (TP) in major clades is supported by different modalities of automixis: central fusion (CF), terminal fusion (TF) and gamete duplication (GD). Recently, the complete loss of males was reported in some populations of the Kalotermitidae *Glyptotermes nakajimai*, in which the mode of thelytoky, either automixis or apomixis, remains to be firmly demonstrated [44]

The evolution of the AQS breeding system requires both the ability of queens to lay viable unfertilized eggs and the propensity of parthenogens to develop into neotenic queens, in known cases via the nymphal stages [2, 6, 40]. Our results demonstrate that thelytokous parthenogenesis leading to parthenogenetic nymphs and neotenic queens may be widespread in the *Termes* group: it has been previously recorded in the genus *Cavitermes* [13], and here *Palmitermes* and *Spinitermes* from the *Cavitermes* lineage, and in *Inquilinitermes* from the sister *Termes* lineage. Phylogenetic relationships among these genera, depicted in Fig. 3, suggest a single origin of parthenogenetic capacities in the *Termes* group, prior to the separation of the *Cavitermes* and the *Termes* lineages, ca. 25 Mya [41]. While in the two closely related sister genera *Cavitermes* and *Palmitermes*, represented by *C. tuberosus* and *P. impostor*, these capacities evolved in emblematic cases of AQS, we cannot conclude that the thelytoky is systematically used to give rise to AQS system in the remaining two studied species, in part due to limited data and in part due to the apparent plasticity of the breeding system in *I. inquilinus*.

We concluded that the gamete duplication is the most likely mechanism of ploidy restoration in the studied genera, as judged from the extensive sampling of *C. tuberosus* and *P. impostor* and the proposed monophyletic origin of parthenogenesis in the *Termes* group. At the same time, gamete duplication is rather rare among animals, and it almost exclusively coincides with the presence of the endosymbiotic bacterium *Wolbachia* [45, 46]. Interestingly, *Wolbachia* was found to infect *C. tuberosus* and *I. inquilinus*, but not *P. impostor* and *S. trispinosus* [47]. It follows that *Wolbachia* might once have played a role in inducing parthenogenesis in the recent ancestor of the *Termes* group. Some species remain capable of thelytoky despite the secondary loss of *Wolbachia*, possibly through the transfer of genes involved in parthenogenesis into the host’s genome [48–50]. Whereas parthenogenesis was conserved in these species’ life history, the interaction between *Wolbachia* and *C. tuberosus* or *I. inquilinus* evolved toward a mutualistic nutritional relationship, linked to their inquiline lifestyle [47]. Only a few studies investigated the cytogenetic mechanisms of thelytoky in the closest relatives of termites, the cockroaches, in which the facultative parthenogenesis is relatively widespread [51–57]. However, also in cockroaches the rather rare automixis with terminal fusion or gamete duplication was proposed in the case of the American cockroach *Periplaneta americana*, beside other mechanisms, such as apomixis in the speckled cockroach *Nauphoeta cinerea* [52, 53, 58].

In contrast to higher termites in general, the reports of neoteny are frequent also in other species and genera within the *Termes* lineage: *Termes hospes* [59], *T. laticornis*, *T. riograndensis* (as *T. saltans*; [26]), and several Australian taxa probably from the same clade: *Xylochomitermes* (as *Termes*) *occidualis*, *X. reductus*, *Hesperotermes infrequens* [60] and *Cristatitermes pineaformis* [61]. At the same time, our extensive sampling of the species from *Termes fatalis* complex, considered as sister to *Inquilinitermes* and sympatric with the species studied here (Fig. 3b, [20, 21]), did not reveal any traces of secondary reproduction; even the largest inspected nests were headed by a pair of primaries, with the queen being highly physogastric. Nevertheless, the parthenogenetic production of female aspirants and neotenics as a safeguard against queen loss and inbreeding, may ultimately be found more widely throughout the phylogeny of the *Termes* group [17], well beyond the few Neotropical species studied as yet. This life trait would be particularly advantageous in inquilines, species that lost the ability of building their own nest and inhabit the unpredictable environment of the host nest, such as *C. tuberosus*, *S. trispinosus* and *I. inquilinus*.

In how many species and genera this capacity of thelytoky evolved into a true AQS breeding system with the queen replacement by parthenogens as a stable element of the life cycle, remains elusive. In established cases of AQS, the main advantage appears to be the expansion of the colony reproductive potential and lifespan, while it would rather mediate an accelerated colony growth and alate production in *S. minutus* [2, 11]. Whatever the selective pressure promoting the evolution of AQS, species with an AQS syndrome usually achieve a wide geographical distribution, i.e. from the west to the east of the tropical rainforests of South America for *C. tuberosus*, *E. neotenicus* and *S. minutus* [10, 11, 13, 23, 62]. As yet, the known distribution of the newly established genus and species *P. impostor* is limited to three sites in French Guiana [21]. As this species has long been misidentified as *Termes* spp., its real distribution area remains elusive. Given the increasing number of reports on thelytokous parthenogenesis as a systematic part of termite reproductive strategies, new discoveries of alternative breeding systems are to be expected not only within the Termitinae, but in termites in general. This has been recently highlighted in *Glyptotermes nakajimai* (Kalotermitidae), the first termite species in which thelytoky has become the exclusive mode of reproduction in some populations, leading to the loss of males from the societies [44].

## Conclusions

We conclude that the combination of sexual and asexual processes in the colony reproduction in higher termites is not restricted to the three isolated cases previously reported. Our observations indicate that in the species-rich *Termes* group, the capacity of thelytokous production of replacement queens is present in at least four genera, and is based on the same cytogenetic mechanism. This suggests that the conditional use of sexual and thelytokous reproductions is a preadaptation common to the whole *Termes* group, which has, in some species, evolved into stable elements of their breeding systems. In the light of these results, the historical idea of a lifelong monogamy of a single reproductive pair, as the key strategy underlying the reproductive success of higher termites, may appear obsolete.

## Methods

### Sampling

We sampled 32 colonies of *P. impostor*, 14 colonies of *S. trispinosus* and 19 colonies of *I. inquilinus* in 2010–2018 in the rainforest of the Petit Saut dam area (N 05.07°, W 52.87°) in French Guiana. Sampling has been performed with the consent of Office National des Fôrets, Direction Régionale de Guyane (French Guiana). Collection information and social composition of all sampled nests are summarized in the (see Additional file 1: Table S1). Most *P. impostor* nests were de novo built underground constructions made of dense and hard soil, sometimes with a minor epigeous part, often found attached to large roots of living or dead standing trees or fallen dead trunks. Nests of *S. trispinosus* were located underground, most often in adapted nest parts of *Neocapritermes taracua* (Termitinae) at the base of *Astrocaryum* palm trees. *Inquilinitermes inquilinus* is an obligatory inquiline species, strictly associated with the nests of another termite, *Constrictotermes cavifrons* (Nasutitermitinae). Its colonies were extracted from peripheral areas of *C. cavifrons* nests, most often from the bottom parts. The nests and nest parts of all collected species were carefully dissected, and specimens from each caste were stored in 100% ethanol and conserved at − 20 °C. The sex of reproductives and nymphs was determined according to the external abdominal sternite configuration under a stereoscope [63].

### Molecular procedures

Eleven nests of *P. impostor*, four nests of *S. trispinosus* and five nests of *I. inquilinus* were selected for genetic analyses (Additional file 1: Table S1).

For *P. impostor*, 345 individuals —five primary queens, six primary kings, 24 nymphoid (nymph-derived) neotenic queens, one ergatoid (worker-derived) neotenic queen, 8 female and 8 male alates, 24 female and 24 male nymphs (stages 1 to 5), 65 female aspirants (subfertile nymph-like preneotenic stage), 88 soldiers and 92 workers— were genotyped at nine microsatellite loci (Additional file 1: Tables S2 and S3). Six of these loci were previously designed for *C. tuberosus* [64] and successfully amplified in *P. impostor* samples (*Ctub*-21, *Ctub*-43, *Ctub*-72, *Ctub*-74, *Ctub*-80 and *Ctub*-84). Three additional loci (*Ctub*-47, *Ctub*-58 and *Ctub*-88; Additional file 1: Table S2) were specifically designed for *P. impostor* based on the *C. tuberosus* microsatellite library and have been deposited in GenBank repository under accessions MK086020 to MK086022.

In *S. trispinosus*, 43 individuals —two primary queens, two primary kings, one nymphoid neotenic queen, two female nymphs, five female aspirants and 31 workers— were genotyped at three microsatellite loci (Additional file 1: Tables S2 and S4). These markers were described for *C. tuberosus* (*Ctub*-42, *Ctub*-72 and *Ctub*-95; [64]) and successfully amplified in *S. trispinosus* samples.

In the case of *I. inquilinus*, 74 individuals —two primary queens, two primary kings, 10 nymphoid neotenic queens, one neotenic king, 12 female nymphs and aspirants, 11 male nymphs and aspirants, and 36 workers— were genotyped at eight microsatellite loci (Additional file 1: Tables S2 and S5). A new library was specifically developed for this species. Total DNA was extracted from heads of 12 workers from one nest (code I12; Additional file 1: Table S1) using a NucleoSpin Tissue kit (Macherey-Nagel), yielding 0.379 μg of total DNA. Preparation of libraries and sequencing on the Illumina MiSeq platform to produce 250 bp paired-end reads was performed by the GenoScreen genomic platform (Lille, France), resulting in 4,889,660 reads. Quality control on raw reads was performed with FastQC *v*0.11.7 [65] and reads were trimmed when average quality per base dropped below 30 with Trimmomatic *v*0.36 [66]. Reads were assembled by pairs using PANDAseq *v*2.11 [67], which provided 3,440,173 sequences, in which microsatellite motifs with amplifying primers were identified in 39,740 (1.16%) sequences using the QDD *v*3.1.2 pipelines [68]. QDD output files were then further cleaned using the following criteria: (*i*) only one microsatellite motif present in the sequence, (*ii*) a minimum of 20 bp distance between the primers and the motif, (*iii*) the primers do not form hairpin and have a content of GC between 40 and 60%, (*iv*) an expected PCR product size of 150–300 bp, and (*v*) motifs have a minimum of 10 repetitions. On this basis, 24 microsatellite loci with di- or trinucleotide repeats motifs were selected. Universal primers were attached to each forward primer in order to efficiently incorporate fluorescent dyes during PCR for multiplexing [69]. Amplification tests were performed on workers from two nests. Amplification in simplex was carried out in 25 μL reactions containing 0.5 μL (2.5 U) MyTaq DNA polymerase (Bioline GmbH, Germany), 5 μL 5x MyTaq Reaction Buffer, 0.25 μL of fluorescent dye (20 μM), 0.25 μL forward and 0.5 μL reverse primers (20 μM each), 1.5 μL of template DNA and PCR-grade water (q.s.). Cycling conditions were as follows: an initial denaturation step at 95 °C for 3 min; 30 cycles of denaturation at 95 °C for 15 s, annealing at 58 °C for 20 s, and extension at 72 °C for 30 s; a final extension step at 72 °C for 10 min. Amplicons were visualized on an ABI3730XL sequencer (Applied Biosystems) and genotypes were obtained using PeakScanner *v*1.0 (Applied Biosystems). From the initial 24 candidate loci, 11 markers amplified successfully in simplex, eight of them were found to be polymorphic and were selected for multiplex development (*Iinq*-03, *Iinq*-04, *Iinq*-07, *Iinq*-12, *Iinq*-14, *Iinq*-15, *Iinq*-16 and *Iinq*-18; Additional file 1: Table S2). These newly developed microsatellite loci have been deposited in GenBank repository under accessions MK092077 to MK092084.

Multiplexes for all three species were designed using Multiplex Manager *v*1.2 [70] and are shown in (see Additional file 1: Table S2). Cycling conditions and genotyping procedures in multiplex for the genetic analyses followed those specified in [64]. Total DNA was extracted from termite heads using a Chelex-based method [71].

### Data analyses

The complete dataset of genotypes used in this study is available as (Additional file 1: Tables S3–S5). Microsatellite polymorphisms were investigated in each species using genotypes of workers. For each species, the microsatellite data were tested for linkage disequilibrium (LD) between loci using log-likelihood ratio statistics and for deviations from Hardy-Weinberg equilibrium (HWE) with GENEPOP on the Web (http://genepop.curtin.edu.au; [72]). To avoid pseudoreplication due to relatedness of individuals within nests, we used one worker per nest and conducted a total of 10 calculations by using a randomly chosen worker in each replicate. Overall significance of tests was obtained by Fisher’s method of combining *p* values. The significance of these tests was adjusted using Bonferroni corrections for multiple tests. The presence of null alleles or scoring errors owing to allele dropout was checked using the software MICRO-CHECKER *v*2.2.3 [73]. For each species and locus, effective number of alleles (*N*_*E*_), observed and expected heterozygosities (*H*_*O*_ and *H*_*E*_) were calculated using GenAlEx *v*6.5 [74]. Inbreeding coefficient (*F*_*IS*_; [75]) were computed using GENEPOP on the Web.

Genotypes of missing founding reproductives were inferred from those of the sterile colony members (soldiers and/or workers), male nymphs, alates or neotenics (if available) when the social composition and genotype combinations were consistent with a simple family (i.e., headed by a primary queen and a primary king). For this purpose, we presumed the sexual origin of the sterile castes and males in all species and colonies. In some colonies, the primary queen was replaced by a group of mature neotenic queens and the genotypes of the colony members were consistent with the extended family model, resulting from the intervention of sexually produced neotenics into reproduction [76]. In these cases, the genotype(s) of the colony founder(s) could not be unambiguously reconstructed.

In order to detect parthenogenesis in the females of the nymphal line (nymphs, aspirants, neotenic queens), we (*i*) screened multilocus genotypes of females from the nymphal line for the number of exhibited genotypes and the presence/absence of alleles that were exclusive to individual parental genotypes, and (*ii*) compared the observed heterozygosity (*H*_*o*_) of workers with that of females from the nymphal line. Because the automixis with gamete duplication, observed in *C. tuberosus*, leads to completely homozygous female parthenogens, we especially focused on the occurrence of fully homozygous females of the nymphal line. For these individuals, we calculated the probability that they were sexually produced based on the known or inferred parental genotypes. In cases where fully homozygous primary queens were observed, we calculated the probability of their sexual origin from the population allelic frequencies computed from all genotyped workers of the considered species. All genetic diversity parameters were calculated using GenAlEx *v*6.5 [74]. All statistical tests were performed using the R software *v*3.1.3 [77].

## Additional file

Additional file 1: **Figure S1.** Photographs of a female ergatoid (worker-derived) neotenic in *Palmitermes impostor*. **Table S1.** Composition of the sampled nests of *P. impostor*, *S. trispinosus* and *I. inquilinus*. **Table S2.** Microsatellite characteristics and PCR multiplexes used in this study. **Table S3.** Genotypes recorded in 11 analyzed colonies of *P. impostor*. **Table S4.** Genotypes recorded in four analyzed colonies of *S. trispinosus*. **Table S5.** Genotypes recorded in five analyzed colonies of *I. inquilinus*. (PDF 1579 kb)

## Data Availability

Newly developed microsatellite loci have been deposited in GenBank repository under accessions MK086020 – MK086022 and MK092077 – MK092084. Additional information is available for this article (see Additional file 1).

## Abbreviations

AQS: Asexual Queen Succession
*F*_*IS*_: Inbreeding coefficient
*H*_*E*_: Expected heterozygosity
*H*_*O*_: Observed heterozygosity
HWE: Hardy-Weinberg equilibrium
LD: Linkage disequilibrium
*N*_*E*_: Effective number of alleles

## References

[1] Matsuura K, Vargo EL, Kawatsu K, Labadie PE, Nakano H, Yashiro T (2009). Queen succession through asexual reproduction in termites. Science..

[2] Matsuura K (2017). Evolution of asexual queen succession system and its underlying mechanisms in termites. J Exp Biol.

[3] Rabeling C, Kronauer DJC (2013). Thelytokous parthenogenesis in eusocial hymenoptera. Annu Rev Entomol.

[4] Matsuura K, Bignell DE, Roisin Y, Lo N (2011). Sexual and asexual reproduction in termites. Biology of Termites: A Modern Synthesis.

[5] Kobayashi K, Miyaguni Y (2016). Facultative parthenogenesis in the Ryukyu drywood termite *Neotermes koshunensis*. Sci Rep.

[6] Nozaki T, Yashiro T, Matsuura K (2018). Preadaptation for asexual queen succession: queen tychoparthenogenesis produces neotenic queens in the termite *Reticulitermes okinawanus*. Insect Soc.

[7] Vargo EL, Labadie PE, Matsuura K (2012). Asexual queen succession in the subterranean termite *Reticulitermes virginicus*. Proc R Soc B.

[8] Luchetti A, Velonà A, Mueller M, Mantovani B (2013). Breeding systems and reproductive strategies in Italian *Reticulitermes* colonies (Isoptera: Rhinotermitidae). Insect Soc.

[9] Dedeine F, Dupont S, Guyot S, Matsuura K, Wang C, Habibpour B (2016). Historical biogeography of *Reticulitermes* termites (Isoptera: Rhinotermitidae) inferred from analyses of mitochondrial and nuclear loci. Mol Phylogenet Evol.

[10] Fougeyrollas R, Dolejšová K, Sillam-Dussès D, Roy V, Poteaux C, Hanus R (2015). Asexual queen succession in the higher termite *Embiratermes neotenicus*. Proc R Soc B.

[11] Fougeyrollas R, Křivánek J, Roy V, Dolejšová K, Frechault S, Roisin Y (2017). Asexual queen succession mediates an accelerated colony life cycle in the termite *Silvestritermes minutus*. Mol Ecol.

[12] Rocha MM (2017). Morales-Corrêa e Castro AC, Cuezzo C, Cancello EM. Phylogenetic reconstruction of Syntermitinae (Isoptera, Termitidae) based on morphological and molecular data. PLoS One.

[13] Fournier D, Hellemans S, Hanus R, Roisin Y (2016). Facultative asexual reproduction and genetic diversity of populations in the humivorous termite *Cavitermes tuberosus*. Proc R Soc B.

[14] Emerson AE (1925). The termites of Kartabo, Bartica District, British Guiana. Zoologica.

[15] Emerson AE (1933). Conditioned behavior among termites (Isoptera). Psyche..

[16] Holmgren N (1906). Studien über südamerikanische Termiten. Zool Jahrb.

[17] Hellemans S, Fournier D, Hanus R, Roisin Y (2017). Secondary queens in the parthenogenetic termite *Cavitermes tuberosus* develop through a transitional helper stage. Evol Dev.

[18] Hellemans S., Fournier D., Hanus R., Roisin Y. (2018). Sex ratio variations among years and breeding systems in a facultatively parthenogenetic termite. Insectes Sociaux.

[19] Inward DJG, Vogler AP, Eggleton P (2007). A comprehensive phylogenetic analysis of termites (Isoptera) illuminates key aspects of their evolutionary biology. Mol Phylogenet Evol.

[20] Kyjaková P, Roy V, Jirošová A, Krasulová J, Dolejšová K, Křivánek J (2017). Chemical systematics of Neotropical termite genera with symmetrically snapping soldiers (Termitidae: Termitinae). Zool J Linnean Soc.

[21] Hellemans S, Bourguignon T, Kyjaková P, Hanus R, Roisin Y (2017). Mitochondrial and chemical profiles reveal a new genus and species of Neotropical termite with snapping soldiers, *Palmitermes impostor* (Termitidae: Termitinae). Invertebr Syst.

[22] Carrijo TF, Cancello EM (2011). *Divinotermes* (Isoptera, Termitidae, Termitinae), a new genus from South America. Sociobiology..

[23] Krishna K, Grimaldi DA, Krishna V, Engel MS (2013). Treatise on the Isoptera of the World. 6. Termitidae (Part Three), Incertae sedis, taxa excluded from Isoptera. Bull Am Mus Nat Hist.

[24] de ARA, Dambros C de S, de MJW (2019). A new termite species of the genus *Dihoplotermes* Araújo (Blattaria, Isoptera, Termitidae) from the Brazilian Amazonian rainforest. Acta Amaz.

[25] Myles TG (1999). Review of secondary reproduction in termites (Insecta: Isoptera) with comments on its role in termite ecology and social evolution. Sociobiology..

[26] Noirot C (1956). Les sexués de remplacement chez les termites supérieurs (Termitidae). Insect Soc.

[27] Carrijo TF (2009). Revisão taxonômica do gênero *Spinitermes* Wasmann, 1897 (Isoptera, Termitidae, Termitinae).

[28] Jones DT, Eggleton P, Bignell DE, Roisin Y, Lo N (2011). Global biogeography of termites: a compilation of sources. Biology of termites: a modern synthesis.

[29] Kobayashi K, Hasegawa E, Yamamoto Y, Kawatsu K, Vargo EL, Yoshimura J (2013). Sex ratio biases in termites provide evidence for kin selection. Nat Commun.

[30] Noirot C, Krishna K, Weesner FM (1970). The nest of termites. Biology of termites.

[31] Bourguignon T, Šobotník J, Lepoint G, Martin JM, Hardy OJ, Dejean A (2011). Feeding ecology and phylogenetic structure of a complex neotropical termite assemblage, revealed by nitrogen stable isotope ratios. Ecol Entomol.

[32] Ferreira da Cunha H, Costa DA, do Espírito Santo Filho K, Silva LO, Brandão D (2003). Relationship between *Constrictotermes cyphergaster* and inquiline termites in the Cerrado (Isoptera: Termitidae). Sociobiology..

[33] Jirošová A, Sillam-Dussès D, Kyjaková P, Kalinová B, Dolejšová K, Jančařík A (2016). Smells like home: chemically mediated co-habitation of two termite species in a single nest. J Chem Ecol.

[34] Florencio DF, Marins A, Rosa CS, Cristaldo PF, Araújo APA, Silva IR (2013). Diet segregation between cohabiting builder and inquiline termite species. PLoS One.

[35] Shellman-Reeve JS, Choe JC, Crespi BJ (1997). The spectrum of eusociality in termites. The evolution of social behavior in insects and arachnids.

[36] Ferreira da Cunha H, Brandão D (2002). Multiple reproductives in nests of the Neotropical termite *Constrictotermes cyphergaster* (Isoptera, Termitidae, Nasutitermitinae ). Rev Bras Entomol.

[37] Scheffrahn RH (2014). *Inquilinitermes johnchapmani*, a new termite (Isoptera: Termitidae: Termitinae) from the llanos of north Central Bolivia. Sociobiology..

[38] Jones SC, La Fage JP, Howard RW (1988). Isopteran sex ratios: phylogenetic trends. Sociobiology..

[39] Nutting WL, Krishna K, Weesner FM (1969). Flight and colony foundation. Biology of Termites, Vol. 1.

[40] Matsuura K, Mizumoto N, Kobayashi K, Nozaki T, Fujita T, Yashiro T (2018). A genomic imprinting model of termite caste determination: not genetic but epigenetic inheritance influences offspring caste fate. Am Nat.

[41] Bourguignon T, Lo N, Šobotník J, Ho SYW, Iqbal N, Coissac É (2017). Mitochondrial phylogenomics resolves the global spread of higher termites, ecosystem engineers of the tropics. Mol Biol Evol.

[42] Bourguignon T, Lo N, Cameron SL, Šobotník J, Hayashi Y, Shigenobu S (2015). The evolutionary history of termites as inferred from 66 mitochondrial genomes. Mol Biol Evol.

[43] Wu LW, Bourguignon T, Šobotník J, Wen P, Liang WR, Li HF (2018). Phylogenetic position of the enigmatic termite family Stylotermitidae (Insecta: Blattodea). Invertebr Syst.

[44] Yashiro T, Lo N, Kobayashi K, Nozaki T, Fuchikawa T, Mizumoto N (2018). Loss of males from mixed-sex societies in termites. BMC Biol.

[45] Suomalainen E, Saura A, Lokki J (1987). Cytology and Evolution in Parthenogenesis.

[46] Ma WJ, Schwander T (2017). Patterns and mechanisms in instances of endosymbiont-induced parthenogenesis. J Evol Biol.

[47] Hellemans S, Kaczmarek N, Marynowska M, Calusinska M, Roisin Y, Fournier D (2019). Bacteriome-associated *Wolbachia* of the parthenogenetic termite *Cavitermes tuberosus*. FEMS Microbiol Ecol.

[48] van der Kooi CJ, Schwander T (2014). Evolution of asexuality via different mechanisms in grass thrips (Thysanoptera: *Aptinothrips*). Evolution..

[49] Feldhaar H, Gross R (2009). Insects as hosts for mutualistic bacteria. Int J Med Microbiol.

[50] Hamilton PT, Hodson CN, Curtis CI, Perlman SJ (2018). Genetics and genomics of an unusual selfish sex ratio distortion in an insect. Curr Biol.

[51] Vershinina AO, Kuznetsova VG (2016). Parthenogenesis in Hexapoda: Entognatha and non-holometabolous insects. J Zool Syst Evol Res.

[52] Corley LS, Blankenship JR, Moore AJ, Moore PJ (1999). Developmental constraints on the mode of reproduction in the facultatively parthenogenetic cockroach *Nauphoeta cinerea*. Evol Dev..

[53] Corley LS, Blankenship JR, Moore AJ (2001). Genetic variation and asexual reproduction in the facultatively parthenogenetic cockroach *Nauphoeta cinerea*: implications for the evolution of sex. J Evol Biol.

[54] Roth LM, Willis ER (1956). Parthenogenesis in cockroaches. Ann Entomol Soc Am.

[55] Katoh K, Iwasaki M, Hosono S, Yoritsune A, Ochiai M, Mizunami M (2017). Group-housed females promote production of asexual ootheca in American cockroaches. Zool Lett.

[56] Parker EDJ, Selander RK, Hudson RO, Lester LJ (1977). Genetic diversity in colonizing parthenogenetic cockroaches. Evolution..

[57] Short JE, Edwards JP (1991). Reproductive and developmental biology of the oriental cockroach *Blatta orientalis* (Dictyoptera). Med Vet Entomol.

[58] Tanaka M, Daimon T (2019). First molecular genetic evidence for automictic parthenogenesis in cockroaches. Insect Sci.

[59] Noirot C (1955). Recherches sur le polymorphisme des termites supérieurs. Ann Sci Nat Zool (11ème série).

[60] Gay FJ (1971). The termitinae (Isoptera) of temperate Australia. Aust J Zool.

[61] Miller LR (1991). A revision of the *Termes-Capritermes* branch of the Termitinae in Australia (Isoptera: Termitidae). Invertebr Taxon.

[62] Krishna K, Grimaldi DA, Krishna V, Engel MS (2013). Treatise on the Isoptera of the World. 4. Termitidae (Part One). Bull Am Mus Nat Hist.

[63] Krishna K, Grimaldi DA, Krishna V, Engel MS (2013). Treatise on the Isoptera of the World. 1. Introduction. Bull Am Mus Nat Hist.

[64] Fournier D, Hanus R, Roisin Y (2015). Development and characterization of microsatellite markers from the humivorous termite *Cavitermes tuberosus* (Isoptera: Termitinae) using pyrosequencing technology. Conserv Genet Resour.

[65] Andrews S (2018). FastQC A quality control tool for high throughput sequence data.

[66] Bolger AM, Lohse M, Usadel B (2014). Trimmomatic: a flexible trimmer for Illumina sequence data. Bioinformatics..

[67] Masella AP, Bartram AK, Truszkowski JM, Brown DG, Neufeld JD (2012). PANDAseq: paired-end assembler for illumina sequences. BMC Bioinformatics.

[68] Meglécz E, Costedoat C, Dubut V, Gilles A, Malausa T, Pech N (2010). QDD: a user-friendly program to select microsatellite markers and design primers from large sequencing projects. Bioinformatics..

[69] Blacket MJ, Robin C, Good RT, Lee SF, Miller AD (2012). Universal primers for fluorescent labelling of PCR fragments-an efficient and cost-effective approach to genotyping by fluorescence. Mol Ecol Resour.

[70] Holleley CE, Geerts PG (2009). Multiplex manager 1.0: a cross-platform computer program that plans and optimizes multiplex PCR. Biotechniques..

[71] Walsh PS, Metzger DA, Higuchi R (1991). Chelex 100 as a medium for simple extraction of DNA for PCR-based typing from forensic material. Biotechniques..

[72] Rousset F (2008). GENEPOP’007: a complete re-implementation of the GENEPOP software for windows and Linux. Mol Ecol Resour.

[73] Van Oosterhout C, Hutchinson WF, Wills DPM, Shipley P (2004). MICRO-CHECKER: software for identifying and correcting genotyping errors in microsatellite data. Mol Ecol Notes.

[74] Peakall R, Smouse PE (2012). GenALEx 6.5: genetic analysis in excel. Population genetic software for teaching and research-an update. Bioinformatics..

[75] Weir B, Cockerham C (1984). Estimating F-statics for the analysis of population structure. Evolution..

[76] Vargo EL, Husseneder C, Grace JK (2003). Colony and population genetic structure of the Formosan subterranean termite, *Coptotermes formosanus*, in Japan. Mol Ecol.

[77] R Development Core Team (2015). R: A Language and Environment for Statistical Computing.

