# Supplementary material for: Widespread occurrence of asexual reproduction in higher termites of the Termes group (Termitidae: Termitinae)
Source: BMC Evol Biol. 2019 Jun 21;19:131. doi: 10.1186/s12862-019-1459-3 (PMC6588926; doi:10.1186/s12862-019-1459-3)
Supplement: Supplementary file 1 — Figure S1. Photographs of a female ergatoid (worker-derived) neotenic in Palmitermes impostor. Table S1. Composition of the sampled nests of P. impostor, S. trispinosus and I. inquilinus. Table S2. Microsatellite characteristics and PCR multiplexes used in this study. Table S3. Genotypes recorded in 11 analyzed colonies of P. impostor. Table S4. Genotypes recorded in four analyzed colonies of S. trispinosus. Table S5. Genotypes recorded in five analyzed colonies of I. inquilinus. (PDF 1579 kb) [file 12862_2019_1459_MOESM1_ESM.pdf]

Widespread occurrence of asexual reproduction in higher termites of the *Termes* group (Termitidae: Termitinae)

Working title: Parthenogenesis in higher termites of the *Termes* group

Simon Hellemans[1,*], Klára Dolejšová[2], Jan Křivánek[2], Denis Fournier[1], Robert Hanus[2,*], Yves Roisin[1]

[1]Evolutionary Biology & Ecology, Université Libre de Bruxelles, Avenue F.D. Roosevelt 50, CP 160/12, B-1050 Brussels, Belgium.

[2]Chemistry of Social Insects, Institute of Organic Chemistry and Biochemistry of the Czech Academy of Sciences, Flemingovo n. 2, CZ-166 10, Prague 6, Czech Republic.

*Corresponding authors: simon.hellemans@ulb.ac.be, robert.hanus@uochb.cas.cz

We dedicate this work to the memory of Philippe Cerdan (1959–2018), the director of HYDRECO laboratory in French Guiana.

ORCID references of authors

Simon Hellemans: 0000-0003-1266-9134; Klára Dolejšová: 0000-0003-4854-2602; Jan Křivánek: 0000-0002-5229-6902; Denis Fournier: 0000-0003-4094-0390; Robert Hanus: 0000-0002-7054-1975; Yves Roisin: 0000-0001-6635-3552

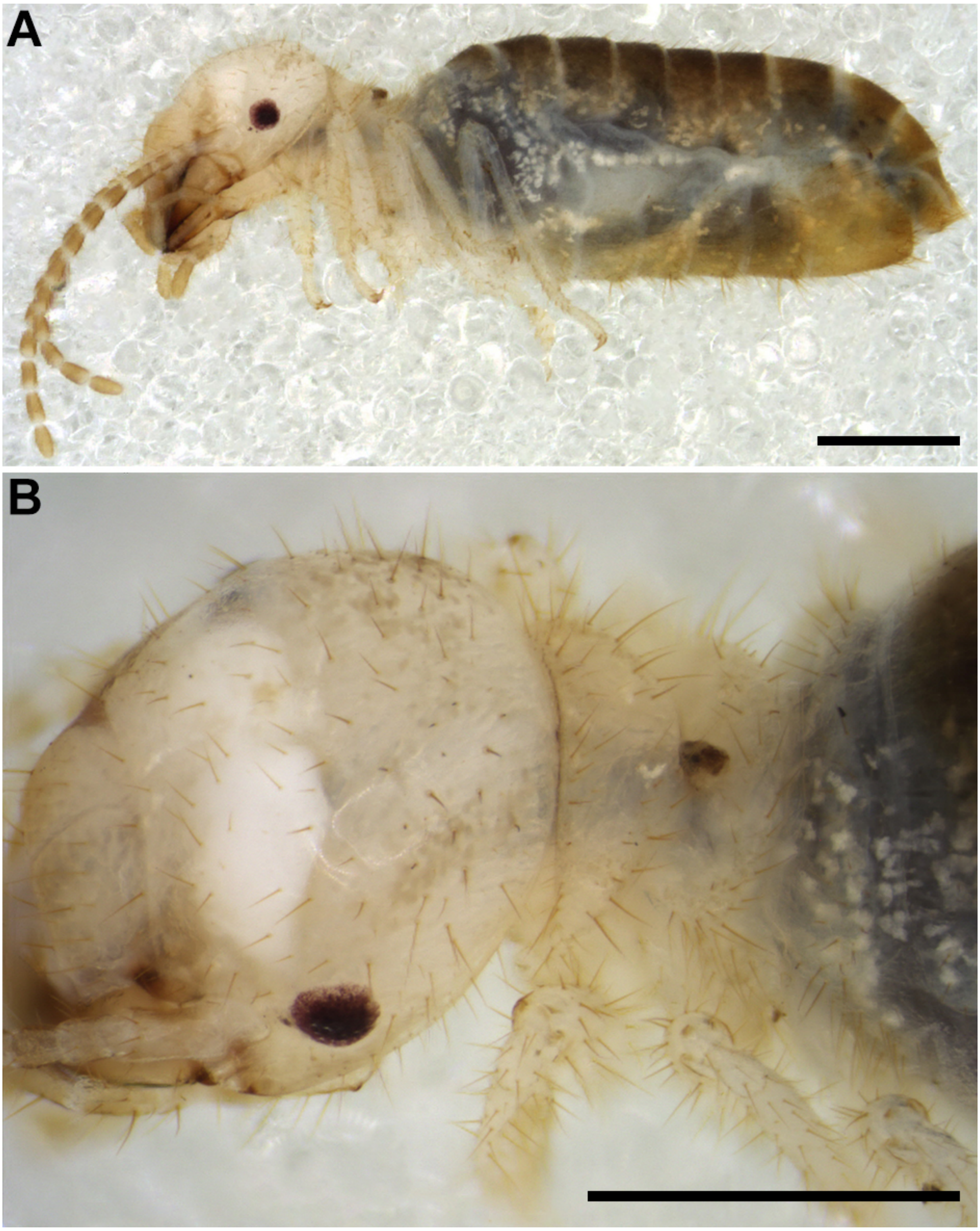

Figure S1. Female ergatoid (worker-derived) neotenic in *Palmitermes impostor* in left lateral view (**A**), and detail of the thorax (**B**) showing fully developed compound eyes and no traces of wingbuds. Images were obtained with a Zeiss Discovery V12 stereomicroscope equipped with an AxioCam ICc3 camera and controlled by AxioVision software. Images are compilations of series of successive stepwise-focused photographs. Scale bars represent 0.5 mm.

Table S1. Composition of the sampled nests of *P. impostor*, *S. trispinosus* and *I. inquilinus*.
Colonies in bold and with colony code were used for genetic analyses

Palmitermes impostor

Colony code	Primary queen	Primary king	Neotenic queens	Female aspirants	Neotenic kings	Nymphs stages 1-4	Nymphs stage 5	Alates	Other repro-ductives	Workers	Soldiers	Collection date	GPS
**PA**	●	●		●		●				●	●	**11/2015**	**N5° 03.980' W52° 59.850'**
**PB**	●	●		●		●				●	●	**11/2015**	**N5° 04.041' W52° 59.873'**
**PC**	●	●		●		●				●	●	**11/2015**	**N5° 03.997' W52° 59.879'**
**PD**	●			●		●				●	●	**11/2015**	**N5° 04.638' W53° 01.325'**
**PE**	●	●		●		●				●	●	**11/2015**	**N5° 04.358' W53° 03.354'**
**PF**				●		●				●	●	**11/2015**	**N5° 04.065' W52° 59.867'**
**PG**				●		●				●	●	**11/2015**	**N5° 04.660' W53° 01.313'**
**PH**	●	●		●		●	●			●	●	**6/2016**	**N5° 04.113' W53° 03.229'**
**PI**			**22**	●		●				●	●	**11/2015**	**N5° 04.290' W52° 58.746'**
**PJ**		●	**98**	●		●			***ergatoid***	●	●	**11/2015**	**N5° 04.237' W52° 58.743'**
**PK**		●	**129**	●		●	●	●	***pseudoimago***	●	●	**6/2016**	**N5° 03.980' W52° 59.875'**
P12	●	●		●		●				●	●	6/2016	N5° 07.187' W52° 57.918'
P13	●	●		●		●				●	●	11/2015	N5° 03.996' W52° 59.889'
P14	●	●		●		●				●	●	6/2016	N5° 04.460' W52° 58.794'
P15	●	●				●				●	●	11/2015	N5° 04.016' W52° 59.914'
P16	●	●		●		●				●	●	6/2016	N5° 04.601' W52° 58.317'
P17	●	●		●		●	●		*ergatoid*	●	●	6/2016	N5° 04.102' W53° 03.075'
P18		●	86	●		●				●	●	6/2016	N5° 04.610' W52° 58.336'
P19			46	●		●	●			●	●	6/2016	N5° 03.975' W52° 59.867'
P20			1	●		●	●			●	●	6/2016	N5° 04.025' W52° 59.876'
P21		●								●	●	11/2015	N5° 03.970' W52° 59.886'
P22				●		●				●	●	11/2015	N5° 03.987' W52° 59.970'
P23				●		●				●	●	6/2016	N5° 04.082' W53° 03.229'
P24				●		●				●	●	6/2016	N5° 04.095' W53° 03.231'
P25				●		●				●	●	11/2015	N5° 03.972' W52° 59.882'
P26						●				●	●	11/2015	N5° 03.925' W52° 59.951'
P27							●			●	●	1/2017	N5° 06.653' W52° 57.944'
P28						●				●	●	4/2017	N5° 03.893' W52° 59.848'
P29										●	●	6/2016	N5° 04.055' W52° 59.872'
P30										●	●	11/2015	N5° 04.201' W52° 58.780'
P31										●	●	4/2017	N5° 03.886' W52° 59.902'
P32										●	●	11/2015	N5° 04.041' W52° 59.873'

Spinitermes trispinosus

Colony code	Primary queen	Primary king	Neotenic queens	Female aspirants	Neotenic kings	Nymphs stages 1-4	Nymphs stage 5	Alates	Other repro-ductives	Workers	Soldiers	Collection date	GPS
**SA**	●	●		●		●				●	●	**6/2016**	**N5° 04.421' W52° 58.776'**
**SB**	●	●		●						●	●	**4/2018**	**N5° 04.401' W52° 58.715'**
**SC**			**1**							●	●	**5/2018**	**N5° 03.941' W52° 58.928'**
**SD**				●		●	●			●	●	**6/2016**	**N5° 04.617' W52° 58.339'**
S5	●	●								●	●	5/2018	N3° 37.194' W53° 11.970'
S6	●	●								●	●	4/2017	N5° 03.891' W52° 59.885'
S7	●	●								●	●	5/2018	N5° 03.912' W52° 58.927'
S8			1			●	●			●	●	4/2017	N5° 04.624' W53° 01.324'
S9										●	●	4/2015	N5° 04.086' W53° 03.241'
S10						●	●			●	●	4/2017	N5° 04.375' W52° 58.767'
S11										●	●	5/2018	N5° 03.917' W52° 58.918'
S12										●	●	5/2018	N3° 37.161' W53° 11.990'
S13										●	●	4/2017	N5° 03.822' W52° 59.919'
S14						●	●			●	●	6/2016	N5° 04.153' W53° 03.311'

Inquilinitermes inquilinus

Colony code	Primary queen	Primary king	Neotenic queens	Female aspirants	Neotenic kings	Nymphs stages 1-4	Nymphs stage 5	Alates	Other repro-ductives	Workers	Soldiers	Collection date	GPS
**IA**				●		●	●			●	●	**4/2018**	**N5° 03.398' W52° 58.659'**
**IB**	**5**	**6**		●		●				●	●	**4/2018**	**N5° 04.454' W52° 58.781'**
**IC**		●	**2**	●	**1**	●	●	●		●	●	**10/2014**	**N5° 04.386' W52° 58.768'**
**ID**	●	●		●		●				●	●	**6/2016**	**N5° 04.335' W52° 58.783'**
**IE**	●	●	**8**	●		●	●			●	●	**4/2018**	**N5° 04.387' W52° 58.687'**
I6	●	●								●	●	4/2018	N5° 04.609' W53° 01.211'
I7	●	●	1	●	2	●	●	●		●	●	5/2018	N3° 37.817' W53° 12.351'
I8		●	3	●		●	●			●	●	4/2018	N5° 04.383' W52° 58.645'
I9	●	●								●		4/2018	N5° 04.226' W53° 02.124'
I10	●	2								●		6/2016	N5° 04.429' W52° 58.761'
I11	8	10								●	●	4/2018	N5° 04.467' W52° 58.778'
I12		●		●		●	●			●	●	4/2017	N5° 03.809' W52° 59.910'
I13			5	●	1	●				●	●	4/2015	N5° 04.335' W52° 58.783'
I14			6							●	●	10/2010	N5° 04.655' W53° 01.309'
I15			10							●	●	3/2014	N5° 04.165' W52° 58.740'
I16						●	●	●		●	●	4/2018	N5° 04.603' W53° 01.208'
I17										●	●	4/2015	N5° 04.423' W53° 03.265'
I18										●	●	9/2012	N5° 04.618' W53° 01.339'
I19										●	●	5/2018	N3° 37.160' W53° 11.980'

Table S2. Microsatellite characteristics and PCR multiplexes used in this study

Palmitermes impostor (92 workers from 11 nests)

Locus [c]	GenBank Accession	5' dye[b]	Repeat motif	Primer sequences (5'-3')	R (bp)	N_A	N_E	f_A (A)	H_O	H_E	F_{IS}
Multiplex P1											
Ctub -72	KJ922359	VIC	(TG)12	F: TGCACTAGTAAGAATATGCACGG<br>R: CGACATCACGTTCATAGCAAG	134-140	3	2.07	0.587 (136)	0.402	0.517	0.227
Ctub -74	KJ922360	VIC	(CTT)13	F: CTGCCTATATTCCACCTTTTTCTT<br>R: ACACGTCGGCGTAAATATCC	90-99	2	1.99	0.527 (99)	0.484	0.498	0.036
Ctub -84	KJ922364	PET	(CA)15	F: GCAAAGAGTAAGAATTATGTCGTTT<br>R: TGTCTGAAATCACGGAGATGA	236-238	2	1.76	0.961 (238)	0.078	0.075	-0.035
Multiplex P2											
Ctub -21	KJ922353	VIC	(AG)10	F: AGCACATGCGAAGTCATCAG<br>R: TCCAGCACAAACATCTTTCA	204-220	2	1.97	0.562 (220)	0.382	0.492	0.230
Ctub -43	KJ922355	VIC	(GT)13	F: ACCCCGATTATGTGAAATGG<br>R: TGAAATTTCTGTACGTGGACCTT	124-128	2	1.61	0.745 (124)	0.380	0.380	0.005
Ctub -80	KJ922363	NED	(AC)15	F: TCTTCGCGATGACAGACACT<br>R: AAACGTTAGTTATGCGGCGA	265-293	7	3.12	0.410 (269)	0.730	0.680	-0.069
Multiplex P3											
Ctub -47 [a]	MK086020	6'FAM (A)	(TG)20	F: AGGTTCCAGTGTGCTGGAGT<br>R: GCGACCAATTTTCTACACCC	133-157	5	3.06	0.473 (133)	0.620	0.673	0.085
Ctub -58 [a]	MK086021	PET (D)	(GT)11	F: TCCATCTTTTAAAGGGAAGCG<br>R: AAGCTGCACAGATGTCAACAC	273-275	2	1.93	0.593 (273)	0.440	0.483	0.095
Ctub -88 [a]	MK086022	VIC (B)	(AC)16	F: TTATCGAACCTGTAGCACGC<br>R: TGGTAGCACGGTAATTCATATACA	171-175	2	1.76	0.685 (171)	0.457	0.432	-0.052

Spinitermes trispinosus (31 workers from 4 nests)

Locus [c]	GenBank Accession	5' dye[b]	Repeat motif	Primer sequences (5'-3')	R (bp)	N_A	N_E	f_A (A)	H_O	H_E	F_{IS}
Multiplex S1											
Ctub -42	KJ922354	NED	(AC)13	F: AGAAGGTGTCATTCAATCATTTG<br>R: GATTCATGACTGCTGATGATTTT	249-253	3	1.58	0.774 (249)	0.452	0.367	-0.216
Ctub -95	KJ922370	PET	(TCT)21	F: GAATCTGAACACAAGTACCCTGC<br>R: TGGTTGAGAAGGCCAAAACT	105-121	5	4.31	0.317 (108)	0.767	0.768	0.018
Multiplex S2											
Ctub -72	KJ922359	VIC	(TG)12	F: TGCACTAGTAAGAATATGCACGG<br>R: CGACATCACGTTCATAGCAAG	132-143	4	3.15	0.448 (132)	0.793	0.683	-0.145

Inquilinitermes inquilinus (36 workers from 5 nests)

Locus [c]	GenBank Accession	5' dye[b]	Repeat motif	Primer sequences (5'-3')	R (bp)	N_A	N_E	f_A (A)	H_O	H_E	F_{IS}
Multiplex I1											
Iinq -03 [a]	MK092077	NED (C)	(AC)19	F: CTTAGATCACACGGCATTCG<br>R: GTATGCCAGCAGTAGCATCG	167-177	4	3.24	0.444 (175)	0.889	0.691	-0.273
Iinq -04 [a]	MK092078	PET (D)	(AG)17	F: TGCGTTGACAAGCTGAGAAG<br>R: AGAACGGTTGGCAATTTAGC	180-182	2	1.36	0.853 (182)	0.294	0.251	-0.158
Iinq -12 [a]	MK092080	PET (D)	(AG)20	F: AACTACTTCCAGCCAAACGG<br>R: GGTGTTGGTGGGTTTACTGC	235-247	6	3.56	0.429 (243)	0.657	0.719	0.101
Iinq -15 [a]	MK092082	NED (C)	(AC)21	F: GTACGATTCTCCGCCTTACG<br>R: TTTACGCTGTTGGAGTTACGTG	255-272	4	3.48	0.371 (255)	0.943	0.712	-0.311
Iinq -18 [a]	MK092084	VIC (B)	(AC)20	F: TTCGTACGTTGAACCCTCAT<br>R: TGGACTCCTATTCAAAGGGAC	311-319	3	1.70	0.742 (315)	0.455	0.413	-0.086
Multiplex I2											
Iinq -07 [a]	MK092079	NED (C)	(AAG)18	F: AGAAACACCGACATGAACCG<br>R: TATTGAAGCGCAATCCCAG	207-226	6	2.68	0.563 (220)	0.844	0.627	-0.332
Iinq -14 [a]	MK092081	VIC (B)	(AC)18	F: AACACGATGTGGTGTCCAAG<br>R: TGCTGCTTCGAATAGTGCAA	259-269	4	2.54	0.574 (259)	0.765	0.606	-0.248
Iinq -16 [a]	MK092083	PET (D)	(AG)20	F: CGGCAGCAGTATAATGGACA<br>R: GCGTAACATCCGAACACTCA	275-283	5	1.89	0.710 (275)	0.581	0.471	-0.218

Allele size range (R), number of alleles (N_A) and effective alleles (N_E), the frequency of the most frequent allele (f_A), observed (H_O) and expected (H_E) heterozygosities, and the inbreeding coefficient (F_{IS}) are based on genotyped workers.

[a] Indicates newly developed microsatellite markers for *P. impostor* from the library prepared for *C. tuberosus* by Fournier *et al.* (2015) and for *I. inquilinus* from the newly developed library.

[b] Fluorescent dyes at the 5' end of the forward primer were either directly integrated during order or incorporated through the three-primer PCR approach developed by Blacket et al. (2012) (used universal primers —tails A, B, C and D— are indicated between parentheses).

[c] Multiplex optimization was carried using Multiplex Manager *v*1.2 (Holleley and Geerts 2009). PCR reagent amounts followed Fournier et al. (2015), and were adapted in the case of loci amplified through the three-primer approach. All multiplexes were amplified following cycling conditions in Fournier et al. (2015).

Table S3. Genotypes recorded in eleven analyzed colonies of *Palmitermes impostor.*
Fully homozygous individuals in bold, genotypes containing alleles exclusive to the mother or one of the inferred parents in red.

Colony	Caste	Locus																	
		Ctub- 21		*Ctub-* 43		*Ctub-* 47		*Ctub-* 58		*Ctub-* 72		*Ctub-* 74		*Ctub-* 80		*Ctub-* 84		*Ctub-* 88	
	(n)	Genotype	*n*	Genotype	*n*	Genotype	*n*	Genotype	*n*	Genotype	*n*	Genotype	*n*	Genotype	*n*	Genotype	*n*	Genotype	*n*
**PA**	primary king	204/220		124/124		153/155		273/273		134/136		90/99		285/291		238/238		171/171	
	primary queen	204/220		124/124		133/153		275/275		136/140		90/90		269/291		238/238		171/175	
	workers	204/220	*5*	124/124	*10*	133/155	*6*	273/275	*10*	134/140	*1*	90/99	*7*	269/285	*4*	238/238	*10*	171/175	*5*
	(10)	220/220	*3*			133/153	*1*			136/136	*6*	90/90	*3*	291/291	*1*			171/171	*5*
		204/204	*2*			153/155	*2*			136/140	*1*			269/291	*3*				
						153/153	*1*			134/136	*2*			285/291	*2*				
	soldiers	204/220	*4*	124/124	*8*	133/155	*5*	273/275	*8*	136/136	*1*	90/99	*5*	291/291	*3*	238/238	*8*	171/175	*5*
	(8)	220/220	*2*			153/155	*2*			136/140	*4*	90/90	*3*	269/291	*2*			171/171	*3*
		204/204	*2*			153/153	*1*			134/136	*3*			285/291	*3*				
	**female aspirant 1**	**220/220**		**124/124**		**133/133**		**275/275**		**140/140**		**90/90**		**291/291**		**238/238**		**175/175**	
	**female aspirant 2**	**204/204**		**124/124**		**133/133**		**275/275**		**136/136**		**90/90**		**291/291**		**238/238**		**171/171**	
	**female aspirant 3**	**220/220**		**124/124**		**153/153**		**275/275**		**136/136**		**90/90**		**291/291**		**238/238**		**175/175**	
	**female aspirant 4**	**220/220**		**124/124**		**133/133**		**275/275**		**140/140**		**90/90**		**291/291**		**238/238**		**175/175**	
	**female aspirant 5**	**220/220**		**124/124**		**133/133**		**275/275**		**136/136**		**90/90**		**269/269**		**238/238**		**171/171**	
	**female aspirant 6**	**220/220**		**124/124**		**153/153**		**275/275**		**136/136**		**90/90**		**291/291**		**238/238**		**171/171**	
	**female aspirant 7**	**220/220**		**124/124**		**153/153**		**275/275**		**140/140**		**90/90**		**269/269**		**238/238**		**171/171**	
	**female aspirant 8**	**220/220**		**124/124**		**133/133**		**275/275**		**136/136**		**90/90**		**291/291**		**238/238**		**171/171**	
**PB**	primary king	204/220		124/128		133/153		273/275		134/136		99/99		269/269		238/238		175/175	
	primary queen	220/220		124/128		133/153		273/275		136/136		99/99		267/291		238/238		171/171	
	workers	204/220	*4*	124/124	*2*	133/153	*2*	273/273	*4*	136/136	*3*	99/99	*8*	267/269	*4*	238/238	*8*	171/175	*8*
	(8)	220/220	*4*	128/128	*1*	153/153	*3*	273/275	*2*	134/136	*5*			269/291	*4*				
				124/128	*5*	133/133	*3*	275/275	*2*										
	soldiers	204/220	*1*	124/124	*4*	133/153	*4*	273/273	*2*	136/136	*7*	99/99	*8*	267/269	*3*	238/238	*8*	171/175	*8*
	(8)	220/220	*7*	128/128	*2*	153/153	*3*	273/275	*5*	134/136	*1*			269/291	*5*				
				124/128	*2*	133/133	*1*	275/275	*1*										
	**female aspirant 1**	**220/220**		**124/124**		**133/133**		**275/275**		**136/136**		**99/99**		**267/267**		**238/238**		**171/171**	
	**female aspirant 2**	**220/220**		**124/124**		**133/133**		**273/273**		**136/136**		**99/99**		**267/267**		**238/238**		**171/171**	
	**female aspirant 3**	**220/220**		**124/124**		**133/133**		**275/275**		**136/136**		**99/99**		**267/267**		**238/238**		**171/171**	
**PC**	primary king	204/204		124/128		155/157		273/273		134/136		90/90		269/291		236/238		171/175	
	primary queen	204/220		124/124		133/155		275/275		134/136		90/90		267/269		238/238		171/175	
	workers	204/204	*3*	124/124	*5*	155/157	*5*	273/275	*10*	134/136	*7*	90/90	*10*	267/291	*4*	236/238	*3*	171/175	*6*
	(10)	204/220	*7*	124/128	*5*	133/157	*3*			136/136	*3*			269/269	*3*	238/238	*7*	171/171	*2*
						155/155	*1*							269/291	*3*			175/175	*2*
						133/155	*1*												
	soldiers	204/204	*5*	124/124	*2*	155/157	*4*	273/275	*8*	134/136	*3*	90/90	*8*	267/291	*2*	236/238	*5*	171/175	*4*
	(8)	204/220	*3*	124/128	*6*	155/155	*1*			134/134	*1*			269/269	*4*	238/238	*3*	171/171	*1*
						133/155	*3*			136/136	*4*			269/291	*1*			175/175	*3*
														267/269	*1*				
	female aspirant 1	204/204		124/128		133/157		273/275		134/134		90/90		267/291		238/238		171/171	
	**female aspirant 2**	**204/204**		**124/124**		**155/155**		**275/275**		**134/134**		**90/90**		**267/267**		**238/238**		**175/175**	
	**female aspirant 3**	**220/220**		**124/124**		**133/133**		**275/275**		**136/136**		**90/90**		**267/267**		**238/238**		**175/175**	
**PD**	primary king (*inferred*)	204/204		124/128		133/155		273/275		134/134		99/99		291/291		238/238		171/175	
	primary queen	204/204		124/124		133/133		275/275		134/140		90/99		269/291		238/238		171/171	
	workers	204/204	*8*	124/124	*2*	133/155	*3*	273/275	*4*	134/134	*5*	90/99	*3*	269/291	*4*	238/238	*8*	171/171	*6*
	(8)			124/128	*6*	133/133	*5*	275/275	*4*	134/140	*3*	99/99	*5*	291/291	*4*			171/175	*2*
	soldiers	204/204	*8*	124/124	*1*	133/155	*2*	273/275	*4*	134/134	*1*	90/99	*3*	269/291	*2*	238/238	*8*	171/171	*4*
	(8)			124/128	*7*	133/133	*6*	275/275	*4*	134/140	*7*	99/99	*5*	291/291	*6*			171/175	*4*
	male nymphs	204/204	*8*	124/128	*3*	133/155	*6*	273/275	*7*	134/134	*5*	90/99	*5*	269/291	*6*	238/238	*8*	171/175	*1*
	(8)			124/124	*5*	133/133	*2*	275/275	*1*	134/140	*3*	99/99	*3*	291/291	*2*			171/171	*7*
	female nymph 1	204/204		124/128		133/133		275/275		134/134		99/99		269/291		238/238		171/171	
	female nymph 2	204/204		124/124		133/133		275/275		134/134		90/99		269/291		238/238		171/175	
	female nymph 3	204/204		124/124		133/133		275/275		134/140		90/99		269/291		238/238		171/171	
	female nymph 4	204/204		124/124		133/155		273/275		134/134		99/99		291/291		238/238		171/175	
	female nymph 5	204/204		124/128		133/155		275/275		134/134		90/99		269/291		238/238		171/171	
	female nymph 6	204/204		124/124		133/155		275/275		134/140		90/99		291/291		238/238		171/171	
	female nymph 7	204/204		124/124		133/155		275/275		134/134		99/99		291/291		238/238		171/171	
	female nymph 8	204/204		124/124		133/133		273/275		134/134		99/99		291/291		238/238		171/171	
	**female aspirant 1**	**204/204**		**124/124**		**133/133**		**275/275**		**140/140**		**90/90**		**291/291**		**238/238**		**171/171**	
	**female aspirant 2**	**204/204**		**124/124**		**133/133**		**275/275**		**140/140**		**90/90**		**269/269**		**238/238**		**171/171**	
	**female aspirant 3**	**204/204**		**124/124**		**133/133**		**275/275**		**140/140**		**90/90**		**269/269**		**238/238**		**171/171**	

Colony	Caste	Locus																	
		Ctub-21		*Ctub*-43		*Ctub*-47		*Ctub*-58		*Ctub*-72		*Ctub*-74		*Ctub*-80		*Ctub*-84		*Ctub*-88	
	(n)	Genotype	*n*	Genotype	*n*	Genotype	*n*	Genotype	*n*	Genotype	*n*	Genotype	*n*	Genotype	*n*	Genotype	*n*	Genotype	*n*
**PE**	primary king	220/220		128/128		133/151		273/273		134/136		90/90		265/293		238/238		171/171	
	primary queen	204/204		124/128		133/133		273/273		134/136		90/99		269/269		236/238		171/171	
	workers	204/220	8	124/128	7	133/151	3	273/273	8	134/136	5	90/99	4	265/269	4	238/238	4	171/171	8
	(8)			128/128	1	133/133	5			136/136	3	90/90	3	269/293	4	236/238	4		
	soldiers	204/220	8	124/128	3	133/151	5	273/273	8	134/136	5	90/99	2	265/269	4	238/238	2	171/171	8
	(8)			128/128	5	133/133	3			136/136	3	90/90	6	269/293	4	236/238	6		
	**female aspirant 1**	**204/204**		**124/124**		**133/133**		**273/273**		**134/134**		**90/90**		**269/269**		**238/238**		**171/171**	
	**female aspirant 2**	**204/204**		**124/124**		**133/133**		**273/273**		**134/134**		**99/99**		**269/269**		**236/236**		**171/171**	
	**female aspirant 3**	**204/204**		**128/128**		**133/133**		**273/273**		**134/134**		**99/99**		**269/269**		**236/236**		**171/171**	
	**female aspirant 4**	**204/204**		**128/128**		**133/133**		**273/273**		**136/136**		**99/99**		**269/269**		**236/236**		**171/171**	
	**female aspirant 5**	**204/204**		**128/128**		**133/133**		**273/273**		**134/134**		**99/99**		**269/269**		**236/236**		**171/171**	
	**female aspirant 6**	**204/204**		**124/124**		**133/133**		**273/273**		**136/136**		**99/99**		**269/269**		**236/236**		**171/171**	
	**female aspirant 7**	**204/204**		**128/128**		**133/133**		**273/273**		**134/134**		**90/90**		**269/269**		**236/236**		**171/171**	
	**female aspirant 8**	**204/204**		**128/128**		**133/133**		**273/273**		**136/136**		**99/99**		**269/269**		**238/238**		**171/171**	
**PF**	parental genotypes	204/220		124/128		133/155		273/273		134/136		90/99		291/291		238/238		171/171	
	(*inferred*)	220/220		124/124		153/153						99/99		267/269				171/175	
	workers	204/220	4	124/124	5	153/155	6	273/273	8	136/136	3	90/99	7	267/291	4	238/238	8	171/175	6
	(8)	220/220	4	124/128	3	133/153	2			134/136	4	99/99	1	269/291	4			171/171	2
										134/134	1								
	soldiers	204/220	8	124/128	5	133/153	3	273/273	8	134/136	4	90/99	6	267/291	3	238/238	8	171/175	5
	(8)			124/124	3	153/155	5			134/134	2	99/99	2	269/291	5			171/171	3
										136/136	2								
	male nymphs	204/220	4	124/124	2	133/153	5	273/273	8	134/136	6	90/99	1	269/291	6	238/238	8	171/175	3
	(8)	220/220	4	124/128	6	153/155	3			134/134	1	99/99	7	267/291	2			171/171	5
										136/136	1								
	female nymph 1	220/220		124/124		153/155		273/273		134/136		99/99		269/291		238/238		171/171	
	female nymph 2	204/220		124/128		153/155		273/273		136/136		90/99		269/291		238/238		171/171	
	female nymph 3	220/220		124/124		133/153		273/273		134/136		99/99		269/291		238/238		171/175	
	female nymph 4	204/220		124/124		133/153		273/273		134/136		99/99		269/291		238/238		171/171	
	female nymph 5	204/220		124/124		153/155		273/273		134/136		99/99		269/291		238/238		171/171	
	female nymph 6	220/220		124/124		133/153		273/273		136/136		99/99		269/291		238/238		171/175	
	female nymph 7	204/220		124/128		153/155		273/273		134/136		99/99		267/291		238/238		171/171	
	female nymph 8	220/220		124/128		133/153		273/273		136/136		99/99		267/291		238/238		171/175	
	**female aspirant 1**	**220/220**		**124/124**		**153/153**		**273/273**		**134/134**		**99/99**		**267/267**		**238/238**		**171/171**	
	**female aspirant 2**	**220/220**		**124/124**		**153/153**		**273/273**		**134/134**		**99/99**		**269/269**		**238/238**		**175/175**	
	**female aspirant 3**	**220/220**		**124/124**		**153/153**		**273/273**		**134/134**		**99/99**		**269/269**		**238/238**		**171/171**	
	**female aspirant 4**	**220/220**		**124/124**		**153/153**		**273/273**		**134/134**		**99/99**		**267/267**		**238/238**		**175/175**	
	**female aspirant 5**	**220/220**		**124/124**		**153/153**		**273/273**		**136/136**		**99/99**		**267/267**		**238/238**		**171/171**	
	**female aspirant 6**	**220/220**		**124/124**		**153/153**		**273/273**		**134/134**		**99/99**		**267/267**		**238/238**		**171/171**	
	**female aspirant 7**	**220/220**		**124/124**		**153/153**		**273/273**		**134/134**		**99/99**		**269/269**		**238/238**		**171/171**	
	**female aspirant 8**	**220/220**		**124/124**		**153/153**		**273/273**		**136/136**		**99/99**		**267/267**		**238/238**		**175/175**	
**PG**	parental genotypes	204/204		124/128		133/157		275/275		134/136		90/90		269/291		238/238		171/175	
	(*inferred*)	204/220		124/124		153/157		273/275				99/99		285/291				171/171	
	workers	204/204	8	124/128	8	133/153	3	275/275	6	136/136	5	90/99	8	291/291	1	238/238	8	171/171	4
	(8)					133/157	3	273/275	2	134/134	1			269/285	5			171/175	4
						153/157	1			134/136	2			269/291	2				
						157/157	1												
	soldiers	204/204	4	124/128	1	153/157	3	275/275	4	136/136	3	90/99	8	291/291	1	238/238	8	171/171	5
	(8)	204/220	4	124/124	7	133/157	2	273/275	4	134/134	3			269/285	2			171/175	3
						157/157	3			134/136	2			269/291	2				
														285/291	2				
	**female aspirant 1**	**204/204**		**128/128**		**153/153**		**273/273**		**134/134**		**99/99**		**291/291**		**238/238**		**171/171**	
	**female aspirant 2**	**204/204**		**128/128**		**157/157**		**273/273**		**136/136**		**99/99**		**291/291**		**238/238**		**171/171**	
	**female aspirant 3**	**204/204**		**128/128**		**157/157**		**275/275**		**136/136**		**99/99**		**291/291**		**238/238**		**171/171**	
	**female aspirant 4**	**220/220**		**128/128**		**157/157**		**275/275**		**134/134**		**99/99**		**285/285**		**238/238**		**171/171**	
	**female aspirant 5**	**220/220**		**128/128**		**157/157**		**273/273**		**136/136**		**99/99**		**285/285**		**238/238**		**171/171**	
	female aspirant 6	204/220		128/128		133/153		275/275		136/136		90/90		291/291		238/238		171/171	
	female aspirant 7	204/220		128/128		133/153		275/275		136/136		90/90		291/291		238/238		171/171	
	**female aspirant 8**	**204/204**		**128/128**		**153/153**		**273/273**		**136/136**		**99/99**		**285/285**		**238/238**		**171/171**	

SUPPORTING INFORMATION

Colony	Caste	Locus																	
		***Ctub*- 21**		***Ctub*- 43**		***Ctub*- 47**		***Ctub*- 58**		***Ctub*- 72**		***Ctub*- 74**		***Ctub*- 80**		***Ctub*- 84**		***Ctub*- 88**	
	**(n)**	**Genotype**	***n***	**Genotype**	***n***	**Genotype**	***n***	**Genotype**	***n***	**Genotype**	***n***	**Genotype**	***n***	**Genotype**	***n***	**Genotype**	***n***	**Genotype**	***n***
**PH**	parental genotypes	220/220		124/124		133/133		275/275		134/136		90/90		287/291		238/238		171/171	
	(*inferred*)	204/220				133/157		273/275		134/140		90/99		269/269					
	workers	204/220	*2*	124/124	*8*	133/157	*4*	273/275	*4*	134/140	*1*	90/90	*5*	269/291	*3*	238/238	*6*	171/171	*8*
	(8)	220/220	*3*			133/133	*4*	275/275	*3*	136/140	*2*	90/99	*2*	269/287	*2*				
										134/136	*2*								
										134/134	*3*								
	soldiers	204/220	*4*	124/124	*8*	133/157	*5*	273/275	*5*	134/140	*1*	90/90	*4*	269/291	*3*	238/238	*8*	171/171	*8*
	(8)	220/220	*3*			133/133	*2*	275/275	*2*	136/140	*4*	90/99	*4*	269/287	*4*				
										134/136	*2*								
										134/134	*1*								
	male nymphs	220/220	*6*	124/124	*8*	133/157	*4*	273/275	*4*	134/140	*2*	90/90	*3*	269/291	*4*	238/238	*8*	171/171	*8*
	(8)	204/220	*2*			133/133	*4*	275/275	*4*	136/140	*1*	90/99	*5*	269/287	*4*				
										134/136	*4*								
										134/134	*1*								
	female nymph 1	204/220		124/124		133/157		273/275		136/140		90/99		269/291		238/238		171/171	
	female nymph 2	220/220		124/124		133/157		273/275		134/136		90/90		269/287		238/238		171/171	
	female nymph 3	220/220		124/124		133/133		273/275		136/140		90/90		269/287		238/238		171/171	
	female nymph 4	220/220		124/124		133/133		275/275		136/140		90/90		269/291		238/238		171/171	
	female nymph 5	220/220		124/124		133/133		275/275		134/140		90/90		269/291		238/238		171/171	
	female nymph 6	220/220		124/124		133/133		275/275		136/140		90/90		269/287		238/238		171/171	
	female nymph 7	204/220		124/124		133/133		273/275		134/140		90/99		269/291		238/238		171/171	
	female nymph 8	204/220		124/124		133/133		275/275		134/134		90/90		269/287		238/238		171/171	
	female aspirant 1	204/220		124/124		133/133		275/275		134/136		90/90		269/291		238/238		171/171	
	**female aspirant 2**	**220/220**		**124/124**		**157/157**		-		**140/140**		**90/90**		**269/269**		**238/238**		**171/171**	
	**female aspirant 3**	**220/220**		**124/124**		**157/157**		**275/275**		**134/134**		**90/90**		**269/269**		**238/238**		**171/171**	
	**female aspirant 4**	**220/220**		**124/124**		**157/157**		**275/275**		**134/134**		**90/90**		**269/269**		**238/238**		**171/171**	
	**female aspirant 5**	**220/220**		**124/124**		**157/157**		**275/275**		**140/140**		**90/90**		**269/269**		**238/238**		**171/171**	
	female aspirant 6	220/220		124/124		133/157		273/275		134/136		90/90		269/291		238/238		171/171	
	female aspirant 7	204/220		124/124		133/157		273/275		134/134		90/99		269/291		238/238		171/171	
	female aspirant 8	220/220		124/124		133/133		273/275		134/140		90/90		269/287		238/238		171/171	
**PI**	workers	220/220	*5*	124/124	*8*	153/153	*3*	273/273	*8*	136/136	*7*	90/99	*5*	291/291	*8*	238/238	*8*	171/175	*5*
	(8)	204/220	*2*			133/153	*5*			134/136	*1*	99/99	*3*					171/171	*2*
		204/204	*1*															175/175	*1*
	soldiers	220/220	*1*	124/124	*8*	133/153	*8*	273/273	*8*	136/136	*5*	90/99	*3*	291/291	*8*	238/238	*8*	171/175	*8*
	(8)	204/220	*4*							134/136	*3*	99/99	*3*						
		204/204	*3*									90/90	*2*						
	neotenic queen 1	220/220		124/124		133/153		273/273		136/136		99/99		291/291		238/238		175/175	
	neotenic queen 2	220/220		124/124		133/153		273/273		136/136		99/99		291/291		238/238		171/175	
	neotenic queen 3	204/220		124/124		153/153		273/273		136/136		90/99		291/291		238/238		175/175	
	neotenic queen 4	204/220		124/124		133/153		273/273		136/136		99/99		291/291		238/238		171/171	
	neotenic queen 5	204/220		124/124		133/133		273/273		134/136		90/99		291/291		238/238		171/171	
	neotenic queen 6	204/220		124/124		133/153		273/273		136/136		90/99		291/291		238/238		171/175	
	**neotenic queen 7**	**220/220**		**124/124**		**153/153**		**273/273**		**136/136**		**99/99**		**291/291**		**238/238**		**175/175**	
	neotenic queen 8	220/220		124/124		153/153		273/273		136/136		90/99		291/291		238/238		171/175	

Colony	Caste	Locus																	
		Ctub- 21		*Ctub*- 43		*Ctub*- 47		*Ctub*- 58		*Ctub*- 72		*Ctub*- 74		*Ctub*- 80		*Ctub*- 84		*Ctub*- 88	
	(n)	Genotype	*n*	Genotype	*n*	Genotype	*n*	Genotype	*n*	Genotype	*n*	Genotype	*n*	Genotype	*n*	Genotype	*n*	Genotype	*n*
**PJ**	primary king	220/220		124/124		133/153		273/275		136/136		90/99		269/291		238/238		171/175	
	workers	220/220	*8*	124/124	*8*	133/153	*4*	273/275	*3*	136/136	*8*	99/99	*8*	269/269	*3*	238/238	*8*	171/175	*3*
	(8)					153/153	*4*	273/273	*4*					269/291	*5*			175/175	*5*
								275/275	*1*										
	soldiers	220/220	*8*	124/124	*8*	133/153	*4*	273/275	*4*	136/136	*8*	99/99	*4*	269/269	*3*	238/238	*8*	171/175	*3*
	(8)					153/153	*4*	273/273	*2*			90/99	*3*	269/291	*5*			175/175	*5*
								275/275	*2*			90/90	*1*						
	**female aspirant 1**	**220/220**		**124/124**		**153/153**		**275/275**		**136/136**		**99/99**		**269/269**		**238/238**		**175/175**	
	**female aspirant 2**	**220/220**		**124/124**		**153/153**		**275/275**		**136/136**		**99/99**		**269/269**		**238/238**		**175/175**	
	**female aspirant 3**	**220/220**		**124/124**		**153/153**		**275/275**		**136/136**		**90/90**		**291/291**		**238/238**		**175/175**	
	**female aspirant 4**	**220/220**		**124/124**		**133/133**		**273/273**		**136/136**		**90/90**		**269/269**		**238/238**		**175/175**	
	**female aspirant 5**	**220/220**		**124/124**		**153/153**		**275/275**		**136/136**		**99/99**		**269/269**		**238/238**		**175/175**	
	**female aspirant 6**	**220/220**		**124/124**		**153/153**		**275/275**		**136/136**		**99/99**		**269/269**		**238/238**		**175/175**	
	**female aspirant 7**	**220/220**		**124/124**		**153/153**		**273/273**		**136/136**		**99/99**		**269/269**		**238/238**		**175/175**	
	female aspirant 8	220/220		124/124		153/153		273/273		136/136		99/99		269/291		238/238		175/175	
	**neotenic queen 1**	**220/220**		**124/124**		**153/153**		**273/273**		**136/136**		**99/99**		**269/269**		**238/238**		**175/175**	
	**neotenic queen 2**	**220/220**		**124/124**		**153/153**		**275/275**		**136/136**		**99/99**		**269/269**		**238/238**		**175/175**	
	**neotenic queen 3**	**220/220**		**124/124**		**153/153**		**275/275**		**136/136**		**99/99**		**269/269**		**238/238**		**175/175**	
	neotenic queen 4	220/220		124/124		133/153		275/275		136/136		90/99		269/269		238/238		175/175	
	**neotenic queen 5**	**220/220**		**124/124**		**153/153**		**275/275**		**136/136**		**99/99**		**269/269**		**238/238**		**175/175**	
	**neotenic queen 6**	**220/220**		**124/124**		**153/153**		**273/273**		**136/136**		**99/99**		**269/269**		**238/238**		**175/175**	
	**neotenic queen 7**	**220/220**		**124/124**		**153/153**		**275/275**		**136/136**		**99/99**		**269/269**		**238/238**		**175/175**	
	neotenic queen 8	220/220		124/124		133/153		273/275		136/136		90/99		269/269		238/238		175/175	
	ergatoid queen	220/220		124/124		153/153		273/275		136/136		99/99		269/269		238/238		171/175	
**PK**	primary king	220/220		128/128		133/133		273/275		134/134		90/99		269/285		238/238		171/175	
	workers	220/220	*6*	124/128	*4*	133/153	*2*	273/275	*5*	134/134	*7*	90/99	*7*	269/285	*1*	238/238	*8*	171/171	*5*
	(8)	204/220	*2*	128/128	*4*	133/133	*5*	275/275	*1*	134/136	*1*	99/99	*1*	267/285	*2*			171/175	*3*
						133/157	*1*	273/273	*2*					269/269	*4*				
														267/269	*1*				
	soldiers	220/220	*6*	124/128	*5*	133/133	*4*	273/275	*4*	134/134	*4*	90/99	*5*	269/285	*2*	238/238	*8*	171/171	*4*
	(8)	204/220	*2*	128/128	*3*	133/157	*4*	275/275	*3*	134/136	*4*	90/90	*2*	267/285	*2*			171/175	*4*
								273/273	*1*			99/99	*1*	269/269	*2*				
														267/269	*2*				
	male alates	220/220	*5*	124/128	*4*	133/133	*3*	273/275	*4*	134/134	*5*	90/99	*4*	267/285	*2*	238/238	*8*	171/171	*5*
	(8)	204/220	*3*	128/128	*4*	133/157	*5*	275/275	*2*	134/136	*3*	90/90	*1*	269/269	*2*			171/175	*3*
								273/273	*2*			99/99	*2*	267/269	*3*				
	female alate 1	204/220		124/128		133/157		273/273		134/134		90/99		-		238/238		171/171	
	female alate 2	204/220		128/128		133/133		273/275		134/134		90/99		267/285		238/238		171/175	
	female alate 3	204/220		124/128		133/133		273/275		134/136		90/99		267/269		238/238		171/175	
	female alate 4	220/220		128/128		133/133		273/275		134/136		90/99		269/269		238/238		171/175	
	female alate 5	204/220		124/128		133/133		273/275		134/136		99/99		269/269		238/238		171/171	
	female alate 6	204/220		128/128		133/133		275/275		134/136		90/99		267/285		238/238		171/175	
	female alate 7	204/220		128/128		133/157		275/275		134/136		90/99		267/285		238/238		171/171	
	female alate 8	220/220		128/128		133/133		273/275		134/136		90/99		267/285		238/238		171/175	
	**female aspirant 1**	**204/204**		**124/124**		**157/157**		**275/275**		**134/134**		**99/99**		**267/267**		**238/238**		**171/171**	
	**female aspirant 2**	**204/204**		**124/124**		**157/157**		**275/275**		**134/134**		**99/99**		**267/267**		**238/238**		**171/171**	
	**female aspirant 3**	**220/220**		**124/124**		**157/157**		**273/273**		**134/134**		**90/90**		**267/267**		**238/238**		**171/171**	
	**female aspirant 4**	**220/220**		**124/124**		**157/157**		**275/275**		**134/134**		**99/99**		**291/291**		**238/238**		**171/171**	
	**female aspirant 5**	**204/204**		**124/124**		**157/157**		**273/273**		**134/134**		**99/99**		**267/267**		**238/238**		**171/171**	
	**female aspirant 6**	**204/204**		**124/124**		**157/157**		**273/273**		**134/134**		**99/99**		**269/269**		**238/238**		**171/171**	
	**female aspirant 7**	**220/220**		**128/128**		**157/157**		**275/275**		**136/136**		**90/90**		**269/269**		**238/238**		**171/171**	
	**female aspirant 8**	**204/204**		**128/128**		**157/157**		**275/275**		**134/134**		**90/90**		**269/269**		**238/238**		**171/171**	
	neotenic queen 1	220/220		124/128		133/153		273/275		136/136		90/99		267/291		238/238		171/175	
	neotenic queen 2	204/220		124/128		133/153		273/275		134/136		90/99		269/291		238/238		171/171	
	neotenic queen 3	220/220		128/128		133/133		273/273		134/134		90/99		267/285		238/238		171/175	
	neotenic queen 4	204/220		124/128		133/157		275/275		134/134		90/99		267/269		238/238		171/171	
	neotenic queen 5	220/220		124/128		133/133		273/275		134/134		90/99		267/285		238/238		171/171	
	neotenic queen 6	204/220		124/128		133/153		273/275		134/136		90/90		269/291		238/238		171/171	
	**neotenic queen 7**	**220/220**		**124/124**		**133/133**		**275/275**		**136/136**		**90/90**		**269/269**		**238/238**		**171/171**	
	neotenic queen 8	204/220		128/128		133/157		275/275		134/136		90/99		267/285		238/238		171/175	

Table S4. Genotypes recorded in four analyzed colonies of *Spinitermes trispinosus.*
Fully homozygous individuals in bold, genotypes containing alleles exclusive to the mother or one of the inferred parents in red.

		locus					
colony	caste	*Ctub*- 42		*Ctub*- 72		*Ctub*- 95	
	(n)	genotype	*n*	genotype	*n*	genotype	*n*
SA	primary king	249/249		138/141		105/105	
	primary queen	249/253		143/143		118/118	
	workers	249/253	*3*	138/143	*3*	105/118	*6*
	(7)	249/249	*4*	141/143	*4*		
	**female aspirant 1**	**253/253**		**143/143**		**118/118**	
SB	primary king	249/253		141/143		108/111	
	**primary queen**	**249/249**		**132/132**		**105/105**	
	workers	249/249	*6*	132/143	*5*	105/108	*7*
	(9)	249/253	*3*	132/141	*2*	105/111	*2*
	female aspirant 1	249/253		132/143		105/108	
	female aspirant 2	249/249		132/143		105/108	
SC	parental genotypes (*inferred*)	249/251		132/143		118/121	
		249/249		132/132		111/118	
	workers	249/249	*5*	132/132	*4*	111/121	*4*
	(8)	249/251	*3*	132/143	*4*	111/118	*1*
						118/121	*1*
						118/118	*2*
	neotenic queen 1	249/251		132/132		111/121	
SD	parental genotypes (*inferred*)	249/253		132/138		108/108	
		249/249				108/111	
	workers	249/249	*2*	132/132	*1*	108/108	*5*
	(7)	249/253	*5*	132/138	*5*	108/111	*2*
				138/138	*1*		
	**female nymph 1**	**249/249**		**132/132**		**108/108**	
	female nymph 2	249/253		132/138		108/108	
	**female aspirant 1**	**249/249**		**132/132**		**108/108**	
	**female aspirant 2**	**249/249**		**132/132**		**108/108**	

Table S5. Genotypes recorded in five analyzed colonies of *Inquilinitermes inquilinus*. Fully homozygous individuals in bold, genotypes containing alleles exclusive to the mother or one of the inferred parents in red.

colony	caste	locus															
		linq- 03		*linq*- 04		*linq*- 07		*linq*- 12		*linq*- 14		*linq*- 15		*linq*- 16		*linq*- 18	
	(n)	genotype	*n*	genotype	*n*	genotype	*n*	genotype	*n*	genotype	*n*	genotype	*n*	genotype	*n*	genotype	*n*
**IA**	parental genotypes	175/177		180/182		220/220		239/241		265/265		270/272		275/283		315/315	
	(*inferred*)	173/177		182/182		214/220		243/245		259/259		268/268		275/275		315/319	
	workers	177/177	*2*	180/182	*5*	220/220	*2*	241/245	*3*	259/265	*7*	268/270	*4*	275/275	*3*	315/315	*5*
	(8)	173/175	*4*	182/182	*3*	214/220	*5*	239/243	*1*			268/272	*4*	275/283	*4*	315/319	*3*
		173/177	*2*					239/245	*3*								
								241/243	*1*								
	**female nymph 1**	**173/173**		**182/182**		**214/214**		**245/245**		**259/259**		**268/268**		**275/275**		**315/315**	
	**female aspirant 1**	**177/177**		**182/182**		**220/220**		**243/243**		**259/259**		**268/268**		**275/275**		**315/315**	
**IB**	primary king	175/177		182/182		220/220		241/247		259/269		255/268		275/275		315/319	
	primary queen	173/173		182/182		207/214		241/241		259/259		270/272		275/283		311/315	
	workers	173/175	*4*	182/182	*4*	207/220	*2*	241/241	*3*	259/259	*1*	255/272	*3*	275/283	*2*	315/315	*1*
	(4)					214/220	*2*	241/247	*1*	259/269	*3*	255/270	*1*	275/275	*2*	311/319	*2*
	male aspirant 1	173/175		182/182		214/220		241/247		259/269		255/270		275/283		315/319	
	male aspirant 2	173/175		182/182		214/220		241/247		259/269		255/270		275/275		311/315	
	male aspirant 3	173/175		182/182		207/220		241/247		259/269		255/272		-		311/319	
	**female aspirant 1**	**173/173**		**182/182**		-		**241/241**		-		**272/272**		-		**315/315**	
	**female aspirant 2**	**173/173**		**182/182**		**214/214**		**241/241**		**259/259**		**272/272**		**283/283**		**315/315**	
**IC**	parental genotypes	167/167		182/182		226/226		235/235		259/269		270/272		277/277		315/319	
	(*inferred*)	175/175				220/220		243/243		259/259		255/255		275/275		315/315	
	workers	167/175	*8*	182/182	*6*	220/226	*5*	235/243	*7*	259/259	*3*	255/270	*5*	275/277	*4*	315/319	*3*
	(8)									259/269	*4*	255/272	*2*			315/315	*3*
	male nymphs	167/175	*3*	182/182	*3*	220/226	*3*	235/243	*3*	259/269	*3*	255/272	*3*	275/277	*3*	315/319	*1*
	(3)															315/315	*2*
	female nymph 1	167/175		182/182		220/226		235/243		259/259		255/270		275/277		315/315	
	female nymph 2	167/175		182/182		220/226		235/243		259/259		255/270		275/277		315/319	
	female nymph 3	167/175		182/182		220/226		235/243		259/259		255/272		275/277		315/315	
	neotenic male	167/175		182/182		220/226		235/243		259/269		255/272		275/277		315/315	
	neotenic queen 1	167/175		182/182		220/226		235/243		259/259		255/270		275/277		315/315	
	**neotenic queen 2**	**175/175**		**182/182**		**220/220**		**243/243**		**259/259**		**255/255**		**275/275**		**315/315**	
**ID**	primary king	173/177		180/182		214/220		243/245		259/269		268/268		275/281		311/315	
	**primary queen**	**175/175**		**182/182**		**220/220**		**243/243**		**259/259**		**255/255**		**275/275**		**315/315**	
	workers	175/177	*2*	182/182	*3*	220/220	*2*	243/243	*3*	259/269	*4*	255/268	*8*	275/281	*2*	315/315	*4*
	(8)	173/175	*6*	180/182	*5*	214/220	*6*	243/245	*5*	259/259	*4*			275/275	*6*	311/315	*4*
**IE**	workers	175/177	*6*	182/182	*8*	220/222	*2*	243/243	*4*	263/265	*3*	255/268	*3*	275/275	*2*	315/315	*5*
	(8)	177/177	*1*			216/220	*4*	241/241	*2*	259/263	*3*	255/270	*2*	275/279	*3*	315/319	*3*
		175/175	*1*			216/222	*1*	241/243	*2*	259/265	*2*	268/270	*1*	275/277	*3*		
						220/220	*1*					268/268	*1*				
												255/255	*1*				
	male nymphs	175/177	*3*	182/182	*5*	216/222	*3*	243/243	*1*	263/265	*1*	255/270	*1*	275/275	*1*	315/315	*1*
	(5)	177/177	*2*			220/220	*2*	241/241	*1*	263/269	*1*	268/268	*4*	277/277	*1*	315/319	*1*
								241/243	*2*	259/265	*2*			271/279	*1*	311/315	*2*
								231/241	*1*	259/269	*1*			277/279	*1*	319/323	*1*
														279/279	*1*		
	female nymph 1	175/177		182/182		216/220		241/243		259/263		255/270		279/279		315/319	
	**female nymph 2**	**177/177**		**182/182**		**216/216**		**243/243**		**259/259**		**270/270**		**279/279**		**319/319**	
	**female nymph 3**	**177/177**		**182/182**		**216/216**		**243/243**		**259/259**		**270/270**		**279/279**		**315/315**	
	female nymph 4	175/177		182/182		220/220		231/241		259/263		268/268		279/279		315/323	
	**female nymph 5**	**175/175**		**182/182**		**220/220**		**243/243**		**263/263**		**255/255**		**279/279**		**315/315**	
	**neotenic queen 1**	**175/175**		**182/182**		**220/220**		**243/243**		**263/263**		**255/255**		**275/275**		**315/315**	
	neotenic queen 2	177/177		182/182		222/222		243/243		263/263		268/268		275/277		315/319	
	neotenic queen 3	177/177		182/182		216/216		241/243		263/263		270/270		275/279		315/319	
	neotenic queen 4	177/177		182/182		216/222		241/241		259/263		268/270		275/279		315/315	
	neotenic queen 5	175/175		182/182		220/220		243/243		259/263		255/255		275/275		315/315	
	**neotenic queen 6**	**177/177**		**182/182**		**216/216**		**243/243**		**263/263**		**270/270**		**275/275**		**315/315**	
	**neotenic queen 7**	**175/175**		**182/182**		**220/220**		**241/241**		**259/259**		**270/270**		**275/275**		**315/315**	
	neotenic queen 8	175/177		182/182		216/220		243/243		259/259		255/270		275/277		315/315	
